# DNA methylation-regulated *ZDHHC5* and *PPT1* in the pathogenesis of osteoporosis

**DOI:** 10.1097/MD.0000000000048429

**Published:** 2026-04-24

**Authors:** Chao Wang, Yong Zhu, Zhe Ruan

**Affiliations:** a Department of Orthopaedics, Xiangya Hospital, Central South University, Changsha, Hunan Province, China; b National Clinical Research Center for Geriatric Disorders, Xiangya Hospital, Central South University, Changsha, Hunan Province, China; c Department of Orthopaedics, The First Hospital of Changsha, Changsha, Hunan Province, China.

**Keywords:** DNA methylation, epigenetic regulation, Mendelian randomization, osteoporosis, palmitoylation

## Abstract

While osteoporosis (OP) affects over 200 million people globally, the causal roles of protein palmitoylation and its upstream epigenetic regulation in the pathogenesis of the disease remain undefined. We aimed to investigate whether DNA methylation causally influences OP risk by modulating the expression of palmitoylation-related genes. We employed an integrated multi-omics causal inference framework, combining 2-sample Mendelian randomization (MR), summary-data-based MR, Bayesian colocalization, and 2-step mediation MR analyses. Data were sourced from large-scale consortia: the FinnGen study (genome-wide association study: 10,461 cases, 473,264 controls), eQTLGen, GTEx (expression quantitative trait loci), and the GoDMC database (methylation quantitative trait loci). Two-sample MR identified *ZDHHC5* as a protective factor (odds ratio = 0.81, 95% confidence interval: 0.76–0.87; *P* = 6.8 × 10^−9^) and the depalmitoylase *PPT1* as a risk factor (odds ratio = 1.06, 95% confidence interval: 1.03–1.08; *P* = 7.9 × 10^−5^) for OP. These findings were corroborated by summary-data-based analysis, and colocalization confirmed a shared causal variant at the *ZDHHC5* locus (posterior probability of H4 = 0.947). Mediation analysis revealed that DNA methylation is a central mechanistic link: methylation at site cg13473383 mediated 92.7% of *ZDHHC5*’s protective effect, while sites cg04560534 and cg07033722 mediated 74.8% and 43.4%, respectively, of *PPT1*’s risk effect. This study is the first to establish a causal epigenetic–palmitoylation axis in OP. The genes *ZDHHC5* and *PPT1*, regulated by specific DNA methylation sites, represent novel potential therapeutic targets and biomarkers, offering fresh insights for precision medicine strategies against bone loss.

## 1. Introduction

Osteoporosis (OP) is a systemic skeletal disorder characterized by compromised bone strength and increased fracture risk, affecting over 200 million people worldwide.^[1]^ With aging populations, OP-related fractures are projected to increase by 33% by 2050, imposing substantial healthcare costs and mortality burdens.^[2]^ The pathogenesis of OP results from an imbalance between osteoblastic bone formation and osteoclastic bone resorption, driven by genetic predisposition, hormonal decline, and epigenetic dysregulation.^[3,4]^ Among various reversible posttranslational modifications, protein palmitoylation has been increasingly recognized as a critical regulator of bone homeostasis.

Palmitoylation is a dynamic lipid modification involving the covalent attachment of 16-carbon palmitate chains to cysteine residues via thioester bonds, thereby conferring hydrophobicity to target proteins and regulating their membrane interactions, intracellular trafficking, and functional stability. This modification cycle is catalyzed by DHHC (aspartic acid–histidine–histidine–cysteine) domain-containing palmitoyltransferases and reversed by palmitoyl-protein thioesterases (e.g., *PPT1*, *PPT2*).^[5,6]^ Studies implicate palmitoylation dysregulation in diverse human diseases, including X-linked intellectual disability caused by *ZDHHC9* mutations^[7]^ and neuronal ceroid lipofuscinosis resulting from *Ppt1* deficiency.^[8]^ In skeletal biology, murine models demonstrate that *Zdhhc13* ablation induces osteopenia through disrupted Wnt/β-catenin signaling.^[9]^ Despite these insights, causal links between palmitoylation genes and OP pathogenesis remain unestablished using genetic epidemiology approaches.

While palmitoylation dynamically regulates bone proteins, DNA methylation represents a complementary epigenetic mechanism involving methyl group addition to cytosine in CpG dinucleotides, catalyzed by DNA methyltransferases. This modification regulates gene expression without altering DNA sequences and is linked to transcriptional repression, genomic imprinting, and cell differentiation.^[10]^ In OP, genome-wide methylation analyses have identified differentially methylated regions,^[11]^ yet whether these changes directly regulate palmitoylation genes to influence bone remodeling is unknown.

Given the established regulatory role of DNA methylation in bone-related gene expression and the emerging significance of palmitoylation in bone homeostasis, we hypothesize that DNA methylation may critically influence OP risk by modulating the expression of key palmitoylation genes. To test this hypothesis, the present study aims to systematically assess the causal relationships between palmitoylation-related genes and OP risk using genetic epidemiology approaches; validate and refine these causal associations by integrating multi-omics data; and quantify the extent to which DNA methylation mechanistically mediates the effects of palmitoylation genes on OP. We address these objectives by employing an integrated analytical framework combining Mendelian randomization (MR), colocalization, and mediation analysis on large-scale genomic and epigenomic datasets.

## 2. Methods

### 2.1. Study design and analytical overview

**Figure 1.**
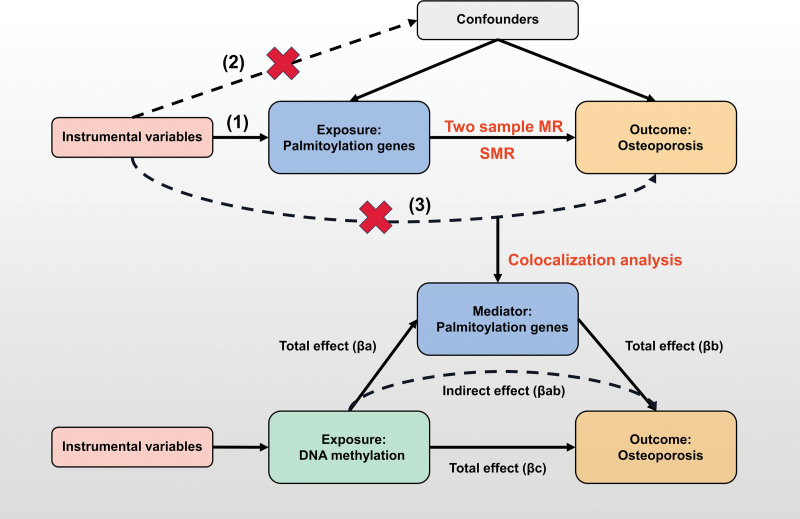
An overview of the study design. MR = Mendelian randomization, SMR = summary-data-based MR.

To address the study objectives, we employed a sequential, multistage causal inference framework using publicly available genetic and epigenetic summary data. The analytical workflow proceeded in 3 principal stages (graphically summarized in Fig. 1). First, we performed a systematic 2-sample MR screen to identify palmitoylation genes exhibiting putative causal associations with OP risk.^[12,13]^ Subsequently, for genes with significant MR associations, we applied summary-data-based MR (SMR) alongside Bayesian colocalization analysis. This step aimed to validate the causal role of gene expression and to evaluate whether the genetic association was driven by shared causal variants, thereby minimizing confounding by linkage disequilibrium (LD).^[14]^ Finally, for the key causal genes identified and validated, we conducted a 2-step, mediation MR analysis to quantify the proportion of their effect on OP that is mediated through specific, instrumented DNA methylation sites.^[15]^ The following subsections detail the data sources, instrumental variable (IV) selection criteria, and specific statistical methods for each stage of the analysis.

### 2.2. Data source

The genome-wide association study (GWAS) summary statistics for OP were obtained from the FinnGen Consortium (R12 release), a population-based study linking genomic data to national health registries in Finland (Table 1). The dataset comprised 10,461 European-ancestry OP cases and 473,264 controls. Case definition adhered to International Classification of Diseases 10th Revision codes M80 (OP with pathological fracture) and M81 (OP without fracture), with exclusions for secondary causes (e.g., M82.0 for glucocorticoid-induced OP). Participants were predominantly female (68.3%) with a median age of 71.2 years. Data were accessed via the FinnGen portal (https://r12.finngen.fi/endpoints/M13_OSTEOPOROSIS).

**Table 1:** Information of included studies and consortia.

Exposure/outcome	Consortium/first author	Participants	Web source
mQTL	GoDMC	27,750 European individuals	http://mqtldb.godmc.org.uk/downloads
eQTL (MR)	eQTLGen Consortium	31,684 individuals (majority of samples were of European ancestry)	https://www.eqtlgen.org/cis-eqtls.html
eQTL (SMR)	GTEx V8	Nearly 1000 deceased individuals	https://gtexportal.org/
Osteoporosis	The FinnGen study	10,461 European-ancestry cases and 473,264 European-ancestry controls	https://r12.finngen.fi/

eQTL = expression quantitative trait loci, mQTL = methylation quantitative trait loci, MR = Mendelian randomization, SMR = summary-data-based MR.

Expression quantitative trait loci (eQTL) data for palmitoylation genes were curated from prior mechanistic studies defining the human palmitoylome, identifying 22 key regulators, including *PPT1*, *PPT2*, and DHHC-family enzymes.^[6,16,17]^ For 2-sample MR, cis-eQTLs of these genes were extracted from the eQTLGen Consortium Phase II (31,684 individuals; 94% European ancestry, whole-blood transcriptomes). For SMR, tissue-specific eQTLs were derived from the GTEx Project V8 (838 postmortem donors across 49 tissues). The complete gene list is provided in Table S1, Supplemental Digital Content, https://links.lww.com/MD/R755. Public access links: eQTLGen (https://www.eqtlgen.org), GTEx (https://gtexportal.org).

Methylation sites information for palmitoylation genes was sourced from https://ngdc.cncb.ac.cn/ewas/datahub/exploration. DNA methylation quantitative trait loci (mQTL) data were acquired from the GoDMC database (Genome-wide mQTL Discovery Project), which aggregated meta-analyses of 420,509 CpG sites across 27,750 European-ancestry individuals. cis-mQTLs for palmitoylation genes (e.g., cg08311476/*ZDHHC5*, cg20955100/*PPT1*) were filtered at a significance threshold of *P* < 1 × 10^−5^. Raw data are available at http://mqtldb.godmc.org.uk/downloads.

### 2.3. Mendelian randomization

The genetic variants utilized as IVs satisfied 3 strict criteria: strong association with the exposure, independence from any modifiable confounders, and independence from any pathway associated with the outcome, except for the exposure pathway.^[18]^ For our MR analysis, we initially extracted IVs based on the genome-wide significance threshold (*P* < 5 × 10^−8^), followed by calculating the *F* statistics of each SNP to exclude weak IVs with *F* statistics < 10. We then ensured independence among IVs for each exposure by conducting an LD test on each SNP identified as an IV based on individuals with European ancestry from the 1000 Genomes Project with *R*^2^ < .001 and a window size of 10,000 kb. If the target SNPs were missing in the outcome GWAS, we used proxy SNPs with high LD (*R*^2^ > 0.8); otherwise, the SNPs were eliminated. In addition, we searched selected SNPs using the LDlink (https://ldlink.nih.gov/) to identify whether they were related to any confounders and outcomes causally associated with OP.^[19]^

To analyze the causal association between genetically predicted palmitoylation genes and OP, we utilized multiple complementary methods, including inverse variance weighted (IVW), MR-Egger, weighted median, simple mode, weighted mode, and Wald ratio methods through TwoSampleMR package. The IVW model was used as the major primary statistical method, and the Wald ratio method was used when a genetic variant contained only 1 genetically related SNP. We assessed the heterogeneity among IVs by using the Cochran *Q* statistic to test, with *P* < .05 indicating heterogeneity. If heterogeneity was present, we used the random-effects model for subsequent analyses; otherwise, we used the fixed-effects model. The MR-PRESSO test was applied to detect outliers in the associations and moderate horizontal pleiotropy by outlier removal.^[20]^ Leave-one-out cross-validation was performed to evaluate the stability of the MR results through sequential exclusion of each IV. In addition, we conducted MR-Egger analysis to identify directional and horizontal pleiotropy.^[21]^ Funnel plot asymmetry indicated the presence of horizontal pleiotropy.^[22]^

### 2.4. SMR analysis

To assess the causal relationship between palmitoylation genes and OP, we performed SMR analysis integrating summary-level data from GWAS and eQTLs.^[14]^ The analysis was conducted using SMR software (version 1.3.1), which infers whether gene expression influences OP risk through shared genetic variation. Given that cis-eQTLs proximal to gene bodies exert more direct transcriptional regulation, we selected lead cis-eQTL variants within ±1 Mb of transcription start sites with minor allele frequency ≥1%. The most significant *cis*-eQTL SNP was selected as a genetic instrument for the target gene. Gene expression effects were quantified as per 1-standard deviation unit change in normalized transcript levels. As SMR assumes associations are driven by single causal variants, we applied the heterogeneity in dependent instruments (HEIDI) test to distinguish true causality from LD confounding. Associations with HEIDI *P* value > .05 were retained, indicating the observed effects were unlikely attributable to LD with neighboring variants. This stringent threshold minimized confounding genetic effects while supporting causal relationships between palmitoylation gene expression and OP risk.

### 2.5. Colocalization analysis

To evaluate whether palmitoylation gene-OP associations share causal genetic variants, we performed Bayesian colocalization analysis using the R package “coloc” (version 5.2.3).^[23]^ For palmitoyltransferase genes demonstrating significant causal effects in MR analysis, we extracted all SNPs within ±100 kb of the lead GWAS variant from summary statistics. Posterior probabilities were computed for competing causal models, where the posterior probability of H4 (PP.H4) quantifies the probability that a single shared variant drives both gene expression and OP associations. Associations with PP.H4 > 0.80 were considered robust evidence for colocalization, reducing false positives and reinforcing MR causality.

### 2.6. Mediation analysis

To determine whether palmitoylation genes functionally mediate the effect of DNA methylation on OP risk, we employed mediation analysis via 2-step MR^[15]^ (Fig. 1) This approach decomposed the total effect of DNA methylation on OP into 2 components: the direct effect of DNA methylation on OP (pathway c), representing effects unmediated by palmitoylation genes; the proportion of mediation was quantified as the ratio of the indirect effect (pathway a × b) to the total effect. Given that mediation analysis assumes no unmeasured confounding, we utilized genetic instruments (cis-mQTLs for methylation, cis-eQTLs for gene expression) to minimize confounding bias. Further sensitivity analyses were conducted to evaluate the robustness of our findings.

## 3. Results

### 3.1. Genetic causality of palmitoylation on OP

We first assessed causal relationships between palmitoylation-related genes and OP risk using 2-sample MR. IVs were derived from genome-wide significant expression quantitative trait loci (*P* < 5 × 10^−8^), yielding 437 independent SNPs after LD clumping and exclusion of variants associated with potential confounders via LDlink. All IVs demonstrated robust strength (*F*-statistics: 29.72–2228.24; mean = 160.39), effectively mitigating weak instrument bias (Table S2, Supplemental Digital Content, https://links.lww.com/MD/R755).

**Figure 2.**
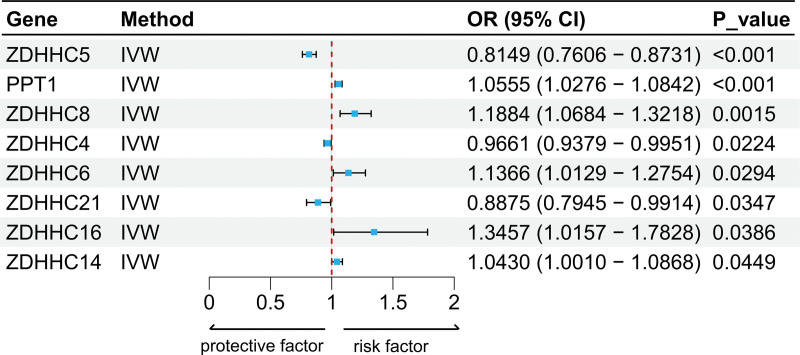
Forest plot of significant MR causal effects of 8 palmitoylation genes on OP based on the IVW method. CI = confidence interval, IVW = inverse variance weighted, MR = Mendelian randomization, OP = osteoporosis, OR = odds ratio.

IVW analysis identified 8 palmitoylation genes significantly associated with OP risk (Fig. 2). Three genes exhibited protective effects: *ZDHHC4* (odds ratio [OR] = 0.97, 95% confidence interval [CI]: 0.94–0.99, *P* = .022), *ZDHHC5* (OR = 0.81, 0.76–0.87, *P* = 6.8 × 10^−9^), and *ZDHHC21* (OR = 0.89, 0.79–0.99, *P* = .035). Five genes showed risk-promoting effects: *ZDHHC6* (OR = 1.14, 1.01–1.28, *P* = .029), *ZDHHC8* (OR = 1.19, 1.07–1.32, *P* = .001), *ZDHHC14* (OR = 1.04, 1.00–1.09, *P* = .045), *ZDHHC16* (OR = 1.35, 1.02–1.78, *P* = .039), and the depalmitoylase *PPT1* (OR = 1.06, 1.03–1.08, *P* = 7.9 × 10^−5^). Consistent directional effects were observed in supplementary MR methods, with SNP-specific estimates detailed in Table S3, Supplemental Digital Content, https://links.lww.com/MD/R755.

Comprehensive sensitivity analyses validated the robustness of causal estimates. Cochran *Q* test detected no significant heterogeneity across instruments (*P* > .05 for all genes), supporting the use of fixed-effect IVW models. MR-Egger regression intercepts approximated null with nonsignificant *P* values (*P* > .05), indicating absence of directional pleiotropy. MR-PRESSO global tests found no evidence of pleiotropic outliers (global *P* > .05; Table S4, Supplemental Digital Content, https://links.lww.com/MD/R755). Leave-one-out analyses demonstrated stable effect estimates upon iterative SNP removal, confirming no single variant drove the associations. Funnel plots exhibited symmetric effect size distributions, further excluding instrument bias (Figs. S1–S8, Supplemental Digital Content, https://links.lww.com/MD/R756).

### 3.2. SMR analysis

**Figure 3.**
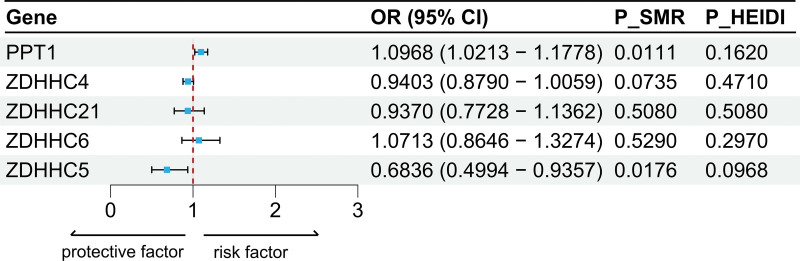
Forest plot of SMR causal effects of palmitoylation-related gene expression on OP. CI = confidence interval, HEIDI = heterogeneity in dependent instruments, OP = osteoporosis, OR = odds ratio, SMR = summary-data-based Mendelian randomization.

To explore the relationship between palmitoylation-related gene expression and OP risk, we implemented SMR. Results demonstrated that solely *ZDHHC5* and *PPT1* showed significant associations with OP in SMR analysis, concordant with primary MR findings (Fig. 3). Specifically, *ZDHHC5* expression levels were inversely correlated with OP risk (β = −0.38, *P* = .018), while *PPT1* expression levels were positively correlated with OP risk (β = 0.092, *P* = .011). To distinguish true causality from LD confounding, we applied the HEIDI test. Nonsignificant HEIDI statistics (*P* = .09 for *ZDHHC5*; *P* = .16 for *PPT1*) indicated that observed associations were unlikely driven by correlated genetic variants, consolidating the robustness of causal inference (Table S5, Supplemental Digital Content, https://links.lww.com/MD/R755).

### 3.3. Colocalization analysis

**Figure 4.**
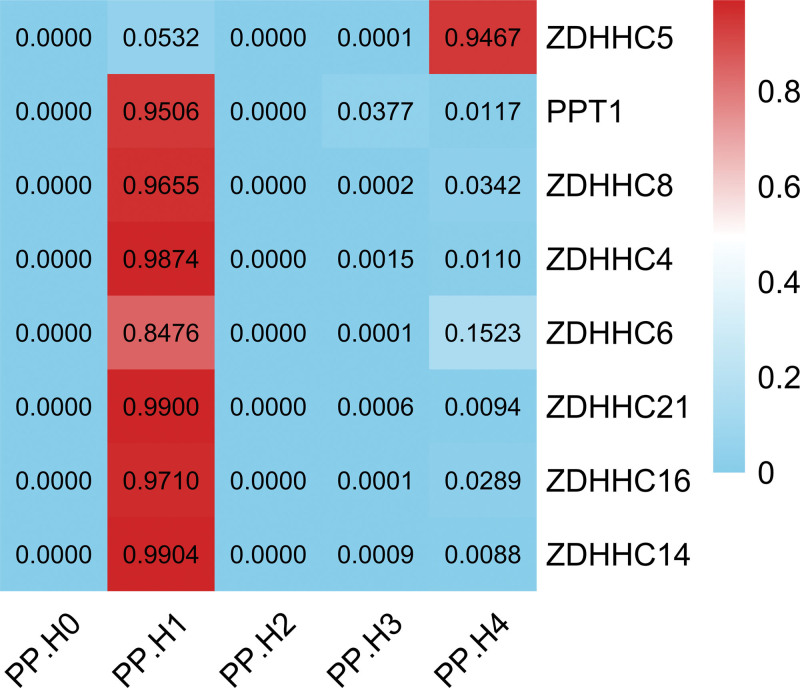
The results of colocalization analysis. PP.H4 quantifies the probability that a single shared variant drives both gene expression and OP associations. A PP.H4 > 0.80 was interpreted as strong evidence supporting colocalization. OP = osteoporosis, PP = posterior probability.

We performed Bayesian colocalization to assess whether genetic associations for palmitoylation genes and OP risk share causal variants at specific genomic loci (Table S6, Supplemental Digital Content, https://links.lww.com/MD/R755). Among all MR-significant genes, only *ZDHHC5* exhibited strong evidence of colocalization with OP (PP.H4 = 0.947), indicating that its association signal is driven by shared causal variants within the *ZDHHC5* locus (Fig. 4). This high posterior probability (PP.H4 > 0.80 threshold) supports intrinsic genetic linkage rather than LD confounding. In contrast, no significant colocalization was observed for other genes. Regional association plots visualize the colocalized signal at chromosome 11q12.1 (Fig. S9, Supplemental Digital Content, https://links.lww.com/MD/R756).

### 3.4. Mediation analysis

To evaluate whether palmitoylation genes (*ZDHHC5*, *PPT1*) mediate the effect of DNA methylation on OP risk, we performed 2-step MR. Methylation site information was retrieved from NGDC, with mQTLs data sourced from the GoDMC database (Table S7, Supplemental Digital Content, https://links.lww.com/MD/R755).

In the first step, we assessed the effect of DNA methylation on gene expression (pathway a, βa). All IVs demonstrated robust strength (*F*-statistics: 37.04–1496.04; mean = 206.66), effectively mitigating weak instrument bias (Table S8, Supplemental Digital Content, https://links.lww.com/MD/R755). IVW analysis identified significant associations: the methylation site cg13473383 was inversely correlated with *ZDHHC5* expression (OR = 0.118, 95% CI: 0.096–0.145, *P* = 1.18 × 10^−93^). For *PPT1*, cg04560534 showed inverse correlation with expression (OR = 0.078, 95% CI: 0.053–0.113, *P* = 5.97 × 10^−41^), while cg07033722 was positively correlated with expression (OR = 9.815, 95% CI: 1.26–76.454, *P* = .029; Table S9, Supplemental Digital Content, https://links.lww.com/MD/R755). In the second step, we evaluated methylation effects on OP risk (pathway c, βc); the same sites demonstrated causal effects: cg13473383 increased risk (OR = 1.60, 95% CI: 1.371–1.872, *P* = 3.03 × 10^−9^), cg04560534 decreased risk (OR = 0.832, 95% CI: 0.758–0.912, *P* = 9.60 × 10^−5^), and cg07033722 increased risk (OR = 1.329, 95% CI: 1.11–1.59, *P* = .001; Tables S10 and S11, Supplemental Digital Content, https://links.lww.com/MD/R755). By integrating these effects with established palmitoylation gene-OP causal relationships (pathway b, βb), we quantified mediation proportions (βa × βb/βc): the methylation site cg13473383 indirectly promoted OP risk by suppressing *ZDHHC5* expression, with *ZDHHC5*-mediated effects accounting for 92.7% of the total effect. For *PPT1*, cg04560534 reduced OP risk by downregulating its expression (74.8%), while cg07033722 increased risk through *PPT1* upregulation (43.4%; Table 2). The robustness of these mediation estimates was supported by sensitivity analyses (Table S12, Supplemental Digital Content, https://links.lww.com/MD/R755).

**Table 2:** Mediator analysis results of DNA methylation sites, *ZDHHC5* or *PPT1* gene expression, and OP disease risk.

Exposure	Outcome	Mediator	Total effect (βc)	βa	βb	Indirect effect (βab)	Proportion mediated
cg13473383	OP	*ZDHHC5*	0.471	−2.135	−0.205	0.437	92.70%
cg04560534	OP	*PPT1*	−0.184	−2.552	0.054	−0.138	74.78%
cg07033722	OP	*PPT1*	0.284	2.284	0.054	0.123	43.39%

OP = osteoporosis.

## 4. Discussion

In this study, our integrated analysis provides the first causal evidence linking DNA methylation to the regulation of palmitoylation genes *ZDHHC5* and *PPT1* in OP. Using large-scale 2-sample MR, SMR validation, colocalization, and mediation analysis, we revealed that *ZDHHC5* exerts a strong protective effect while *PPT1* promotes OP risk. Notably, methylation sites contribute substantially to OP risk by modulating these gene expressions, establishing an epigenetic–palmitoylation regulatory axis critical for bone homeostasis.

Our findings suggest that elevated *ZDHHC5* expression protects against OP, consistent with its established role in palmitoylation and membrane protein trafficking. Recent studies demonstrate that *ZDHHC5*-mediated palmitoylation of NOD2 enhances osteoblast differentiation and mineralization.^[24]^ In senile OP models, *ZDHHC5* upregulation rejuvenated bone marrow mesenchymal stem cells and mitigated bone loss.^[25]^ These experimental observations echo prior reviews emphasizing DHHC enzymes’ roles in osteoblast function and skeletal maintenance.^[6]^ Although direct in vivo knockout models for *ZDHHC5* are limited, evidence from related DHHC family members, such as *Zdhhc13*, reinforces the family’s importance in bone biology.^[9]^

We further identified *PPT1* as a risk-promoting gene in OP. *PPT1* encodes a depalmitoylating enzyme that removes palmitate residues, influencing protein turnover and lysosomal function.^[26]^ Its expression in bone marrow and bone tissue suggests functions beyond classical neuronal roles.^[27]^ Experimental studies show that *Ppt1* deficiency disrupts lysosomal homeostasis, potentially impairing osteoblast and osteoclast activity.^[27,28]^ A recent methylome-transcriptome study in aged murine bone further implicates *Ppt1* in bone metabolism pathways,^[28]^ supporting our causal inference.

Beyond the genetic causality of palmitoylation genes, our study provides novel evidence that DNA methylation critically mediates OP risk by regulating *ZDHHC5* and *PPT1* expression. Notably, mediation analysis indicated exceptionally high proportions of indirect effects – for example, cg13473383 explaining ~92.7% of *ZDHHC5*’s protective effect – underscoring methylation as a key upstream regulator of palmitoylation balance. This aligns with prior epigenome-wide studies showing age- and disease-related CpG methylation shifts in bone tissues, affecting osteoblast and osteoclast gene programs.^[10,29]^ Importantly, the identified CpG sites (e.g., cg13473383, cg04560534) localize within promoter-proximal regions or gene bodies of palmitoylation genes, suggesting tissue-specific transcriptional control. Such methylation patterns are functionally relevant: *ZDHHC5* expression inversely correlates with methylation, consistent with classic CpG island hypermethylation-induced silencing.^[10]^ Conversely, *PPT1* expression shows both positive and negative correlations, reflecting complex regulation likely influenced by chromatin context and enhancer methylation.^[6]^ Clinically, these findings highlight the therapeutic potential of targeting epigenetic modifiers to restore protective palmitoylation gene expression or suppress risk genes like *PPT1*. Recent advances in locus-specific epigenome editing (e.g., dCas9-TET1, dCas9-DNMT3A) enable precise methylation remodeling, offering hope for disease modification.^[30]^ Together, our results establish a causal epigenetic–palmitoylation axis in OP pathogenesis, bridging genetic epidemiology and molecular biology toward translational opportunities.

This study has several strengths. First, by integrating 2-sample MR, SMR validation, colocalization, and mediation analysis, we constructed a comprehensive multilayered causal inference framework, thus overcoming limitations of conventional observational studies prone to confounding and reverse causation.^[12]^ Second, large-scale, high-quality summary statistics from FinnGen, eQTLGen, GTEx, and GoDMC consortia provided robust statistical power and reproducibility while leveraging tissue-wide and methylome-wide data. Third, our study is, to our knowledge, the first to delineate an epigenetic–palmitoylation regulatory axis in OP, integrating genetic, transcriptomic, and epigenetic data. Nevertheless, certain limitations warrant caution. Our analyses were predominantly based on European-ancestry cohorts; thus, generalizability to other ethnic groups remains to be validated. Second, although we used cis-eQTLs and cis-mQTLs to infer tissue-relevant effects, current datasets largely derive from blood rather than bone-specific samples, which may introduce tissue heterogeneity.^[21]^ Third, MR assumptions – particularly the absence of horizontal pleiotropy – cannot be fully excluded, although our sensitivity analyses (e.g., MR-Egger, HEIDI, MR-PRESSO) suggested robustness.^[20]^

Future research should validate these findings through experimental perturbation of CpG methylation and palmitoylation gene expression in bone cells and animal models. Single-cell multi-omics in osteoblasts and osteoclasts could elucidate cell-type-specific regulatory mechanisms, while emerging CRISPR/dCas9-based epigenetic editing tools offer therapeutic promise to modulate risk loci.^[31]^ Overall, our integrative approach paves the way for identifying novel biomarkers and developing precision epigenetic interventions for OP.

## 5. Conclusion

In summary, this integrative study demonstrates that DNA methylation significantly modulates OP risk by regulating the expression of palmitoylation genes *ZDHHC5* and *PPT1*. MR and mediation analyses provide robust evidence for a causal epigenetic–palmitoylation axis underlying bone fragility. These findings highlight *ZDHHC5* as a protective factor and *PPT1* as a risk gene, offering novel insights into the molecular pathogenesis of OP and suggesting promising targets for epigenetic-based intervention strategies.

## Author contributions

**Conceptualization:** Chao Wang, Zhe Ruan.

**Data curation:** Chao Wang, Zhe Ruan.

**Formal analysis:** Chao Wang.

**Software:** Chao Wang.

**Validation:** Chao Wang, Zhe Ruan.

**Visualization:** Chao Wang, Zhe Ruan.

**Funding acquisition:** Yong Zhu.

**Methodology:** Yong Zhu, Zhe Ruan.

**Supervision:** Yong Zhu.

**Writing – original draft:** Zhe Ruan.

**Writing – review & editing:** Chao Wang, Yong Zhu, Zhe Ruan.

## Supplementary Material





## References

[1] Clynes MA Harvey NC Curtis EM Fuggle NR Dennison EM Cooper C. The epidemiology of osteoporosis. Br Med Bull. 2020;133:105–17. 32282039 10.1093/bmb/ldaa005 PMC7115830

[2] Rashki Kemmak A Rezapour A Jahangiri R Nikjoo S Farabi H Soleimanpour S. Economic burden of osteoporosis in the world: a systematic review. Med J Islam Repub Iran. 2020;34:154. 33437750 10.34171/mjiri.34.154 PMC7787041

[3] Compston JE McClung MR Leslie WD. Osteoporosis. Lancet. 2019;393:364–76. 30696576 10.1016/S0140-6736(18)32112-3

[4] Zhu X Bai W Zheng H. Twelve years of GWAS discoveries for osteoporosis and related traits: advances, challenges and applications. Bone Res. 2021;9:23. 33927194 10.1038/s41413-021-00143-3 PMC8085014

[5] Linder ME Deschenes RJ. Palmitoylation: policing protein stability and traffic. Nat Rev Mol Cell Biol. 2007;8:74–84. 17183362 10.1038/nrm2084

[6] Chamberlain LH Shipston MJ. The physiology of protein S-acylation. Physiol Rev. 2015;95:341–76. 25834228 10.1152/physrev.00032.2014 PMC4551212

[7] Raymond FL Tarpey PS Edkins S. Mutations in ZDHHC9, which encodes a palmitoyltransferase of NRAS and HRAS, cause X-linked mental retardation associated with a Marfanoid habitus. Am J Hum Genet. 2007;80:982–7. 17436253 10.1086/513609 PMC1852737

[8] Mondal A Appu AP Sadhukhan T. Ppt1-deficiency dysregulates lysosomal Ca(++) homeostasis contributing to pathogenesis in a mouse model of CLN1 disease. J Inherit Metab Dis. 2022;45:635–56. 35150145 10.1002/jimd.12485 PMC9090967

[9] Saleem AN Chen YH Baek HJ. Mice with alopecia, osteoporosis, and systemic amyloidosis due to mutation in Zdhhc13, a gene coding for palmitoyl acyltransferase. PLoS Genet. 2010;6:e1000985. 20548961 10.1371/journal.pgen.1000985 PMC2883605

[10] Jones PA. Functions of DNA methylation: islands, start sites, gene bodies and beyond. Nat Rev Genet. 2012;13:484–92. 22641018 10.1038/nrg3230

[11] Delgado-Calle J Sañudo C Bolado A. DNA methylation contributes to the regulation of sclerostin expression in human osteocytes. J Bone Miner Res. 2012;27:926–37. 22162201 10.1002/jbmr.1491

[12] Davies NM Holmes MV Smith GD. Reading Mendelian randomisation studies: a guide, glossary, and checklist for clinicians. BMJ. 2018;362:k601. 30002074 10.1136/bmj.k601 PMC6041728

[13] Smith GD Ebrahim S. “Mendelian randomization”: can genetic epidemiology contribute to understanding environmental determinants of disease? Int J Epidemiol. 2003;32:1–22. 12689998 10.1093/ije/dyg070

[14] Zhu Z Zhang F Hu H. Integration of summary data from GWAS and eQTL studies predicts complex trait gene targets. Nat Genet. 2016;48:481–7. 27019110 10.1038/ng.3538

[15] Carter AR Sanderson E Hammerton G. Mendelian randomisation for mediation analysis: current methods and challenges for implementation. Eur J Epidemiol. 2021;36:465–78. 33961203 10.1007/s10654-021-00757-1 PMC8159796

[16] Li M Zhang L Chen CW. Diverse roles of protein palmitoylation in cancer progression, immunity, stemness, and beyond. Cells. 2023;12:2209. 37759431 10.3390/cells12182209 PMC10526800

[17] Zhou B Hao Q Liang Y Kong E. Protein palmitoylation in cancer: molecular functions and therapeutic potential. Mol Oncol. 2023;17:3–26. 36018061 10.1002/1878-0261.13308 PMC9812842

[18] Burgess S Smith GD Davies NM. Guidelines for performing Mendelian randomization investigations: update for summer 2023. Wellcome Open Res. 2019;4:186. 32760811 10.12688/wellcomeopenres.15555.1 PMC7384151

[19] Machiela MJ Chanock SJ. LDlink: a web-based application for exploring population-specific haplotype structure and linking correlated alleles of possible functional variants. Bioinformatics. 2015;31:3555–7. 26139635 10.1093/bioinformatics/btv402 PMC4626747

[20] Verbanck M Chen CY Neale B Do R. Detection of widespread horizontal pleiotropy in causal relationships inferred from Mendelian randomization between complex traits and diseases. Nat Genet. 2018;50:693–8. 29686387 10.1038/s41588-018-0099-7 PMC6083837

[21] Burgess S Thompson SG. Interpreting findings from Mendelian randomization using the MR-Egger method. Eur J Epidemiol. 2017;32:377–89. 28527048 10.1007/s10654-017-0255-x PMC5506233

[22] Hemani G Zheng J Elsworth B. The MR-Base platform supports systematic causal inference across the human phenome. Elife. 2018;7:e34408. 29846171 10.7554/eLife.34408 PMC5976434

[23] Giambartolomei C Vukcevic D Schadt EE. Bayesian test for colocalisation between pairs of genetic association studies using summary statistics. PLoS Genet. 2014;10:e1004383. 24830394 10.1371/journal.pgen.1004383 PMC4022491

[24] Wang X Zhang Y Lin Z Wang H Xu G Ma X. The role of palmitoylation modifications in the regulation of bone cell function, bone homeostasis, and osteoporosis. Bone Joint Res. 2025;14:420–33. 40341006 10.1302/2046-3758.145.BJR-2024-0259.R2 PMC12061513

[25] Tian RC Zhang RY Ma CF. Rejuvenation of bone marrow mesenchymal stem cells: mechanisms and their application in senile osteoporosis treatment. Biomolecules. 2025;15:276. 40001580 10.3390/biom15020276 PMC11853522

[26] Gupta P Soyombo AA Atashband A. Disruption of PPT1 or PPT2 causes neuronal ceroid lipofuscinosis in knockout mice. Proc Natl Acad Sci U S A. 2001;98:13566–71. 11717424 10.1073/pnas.251485198 PMC61081

[27] Dearborn JT Ramachandran S Shyng C. Histochemical localization of palmitoyl protein thioesterase-1 activity. Mol Genet Metab. 2016;117:210–6. 26597320 10.1016/j.ymgme.2015.11.004 PMC4755911

[28] Suopanki J Partanen S Ezaki J Baumann M Kominami E Tyynelä J. Developmental changes in the expression of neuronal ceroid lipofuscinoses-linked proteins. Mol Genet Metab. 2000;71:190–4. 11001810 10.1006/mgme.2000.3071

[29] Lin Y Jiang S Yao Y. Posttranslational modification in bone homeostasis and osteoporosis. MedComm (2020). 2025;6:e70159. 40170748 10.1002/mco2.70159 PMC11959162

[30] Stepper P Kungulovski G Jurkowska RZ. Efficient targeted DNA methylation with chimeric dCas9-Dnmt3a-Dnmt3L methyltransferase. Nucleic Acids Res. 2017;45:1703–13. 27899645 10.1093/nar/gkw1112 PMC5389507

[31] Hilton IB D’Ippolito AM Vockley CM. Epigenome editing by a CRISPR-Cas9-based acetyltransferase activates genes from promoters and enhancers. Nat Biotechnol. 2015;33:510–7. 25849900 10.1038/nbt.3199 PMC4430400

