# Supplementary material for: DNA methylation-regulated ZDHHC5 and PPT1 in the pathogenesis of osteoporosis
Source: Medicine (Baltimore). 2026 Apr 24;105(17):e48429. doi: 10.1097/MD.0000000000048429 (PMC13124397; doi:10.1097/MD.0000000000048429)

## Slide 1
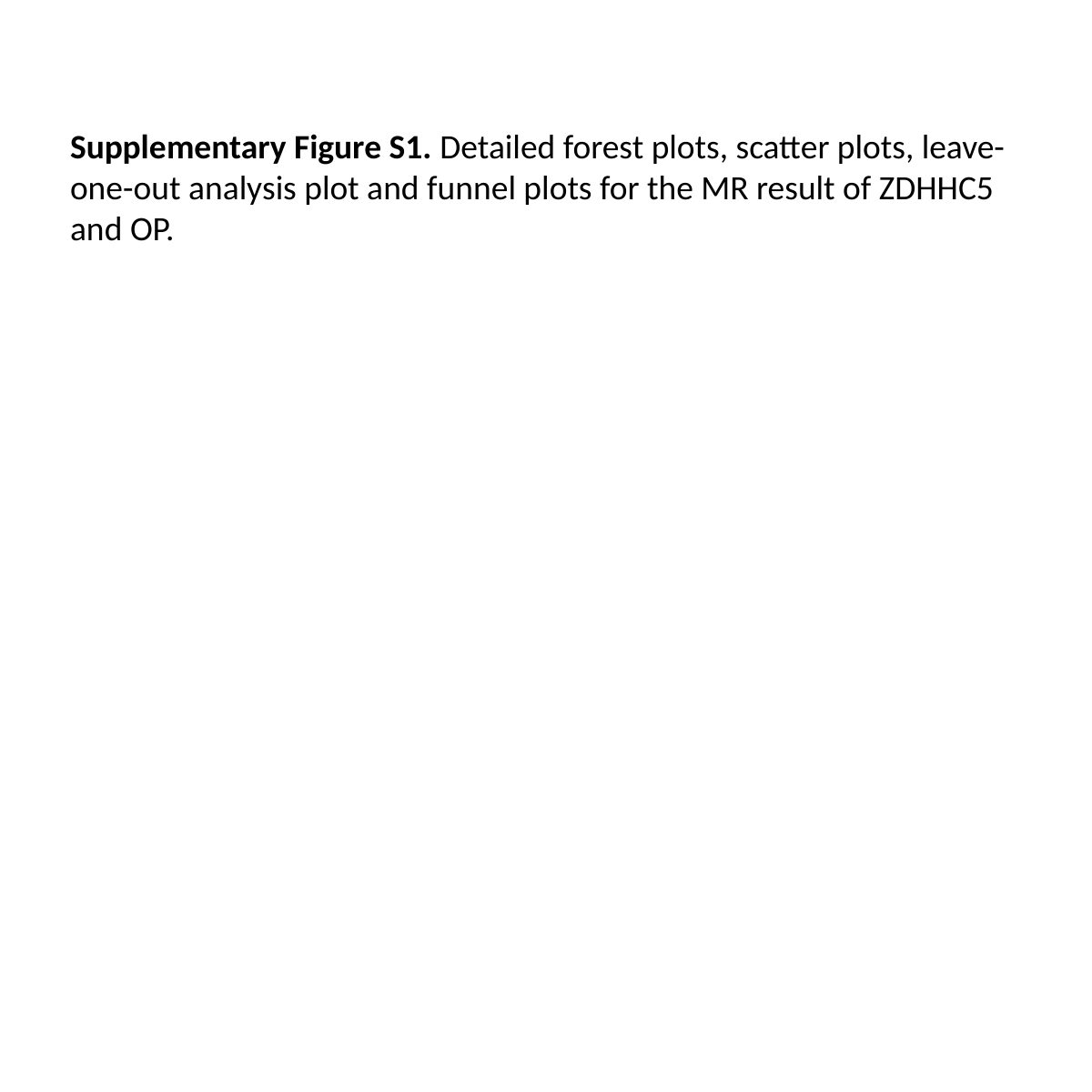

Supplementary Figure S1. Detailed forest plots, scatter plots, leave-one-out analysis plot and funnel plots for the MR result of ZDHHC5 and OP.

## Slide 2
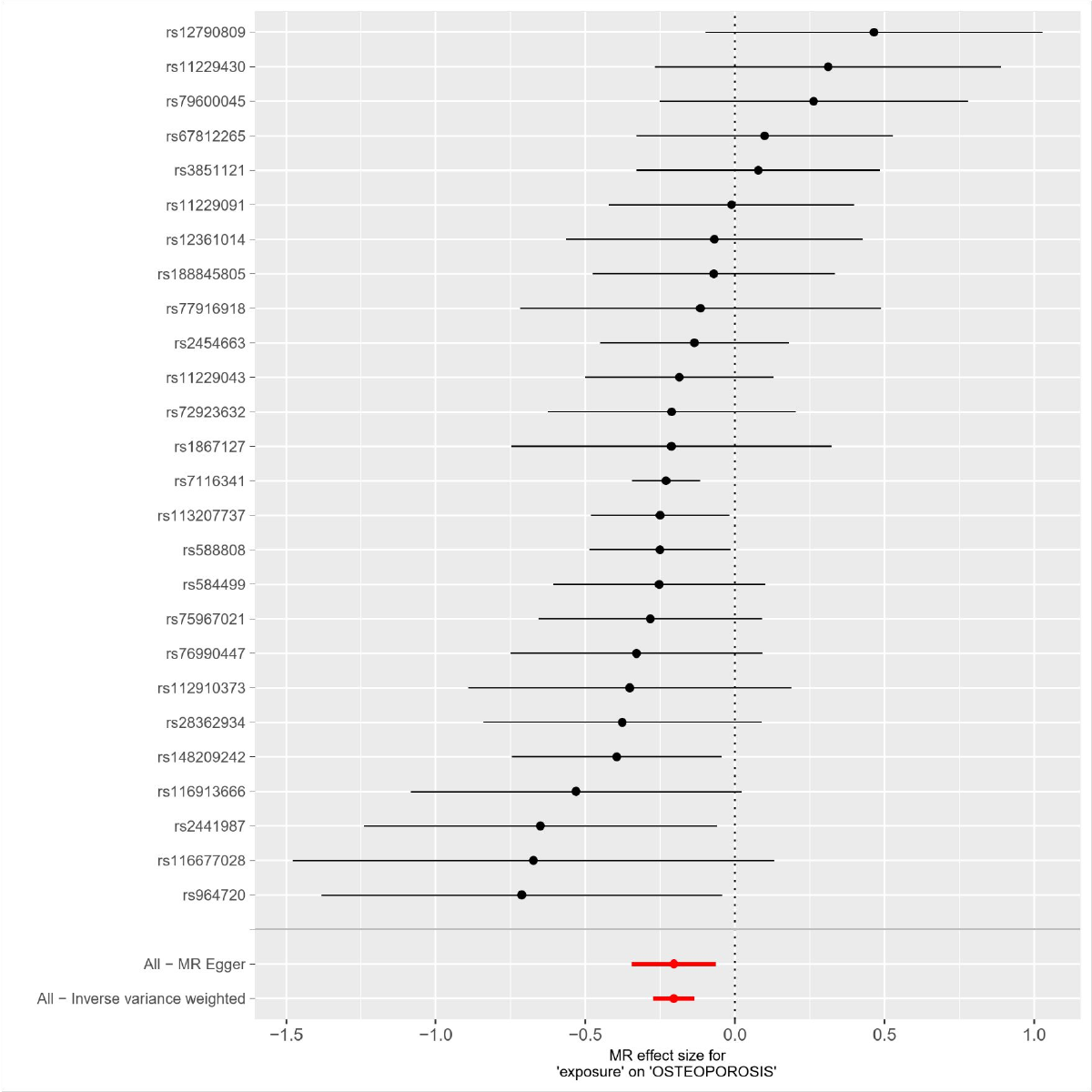

## Slide 3
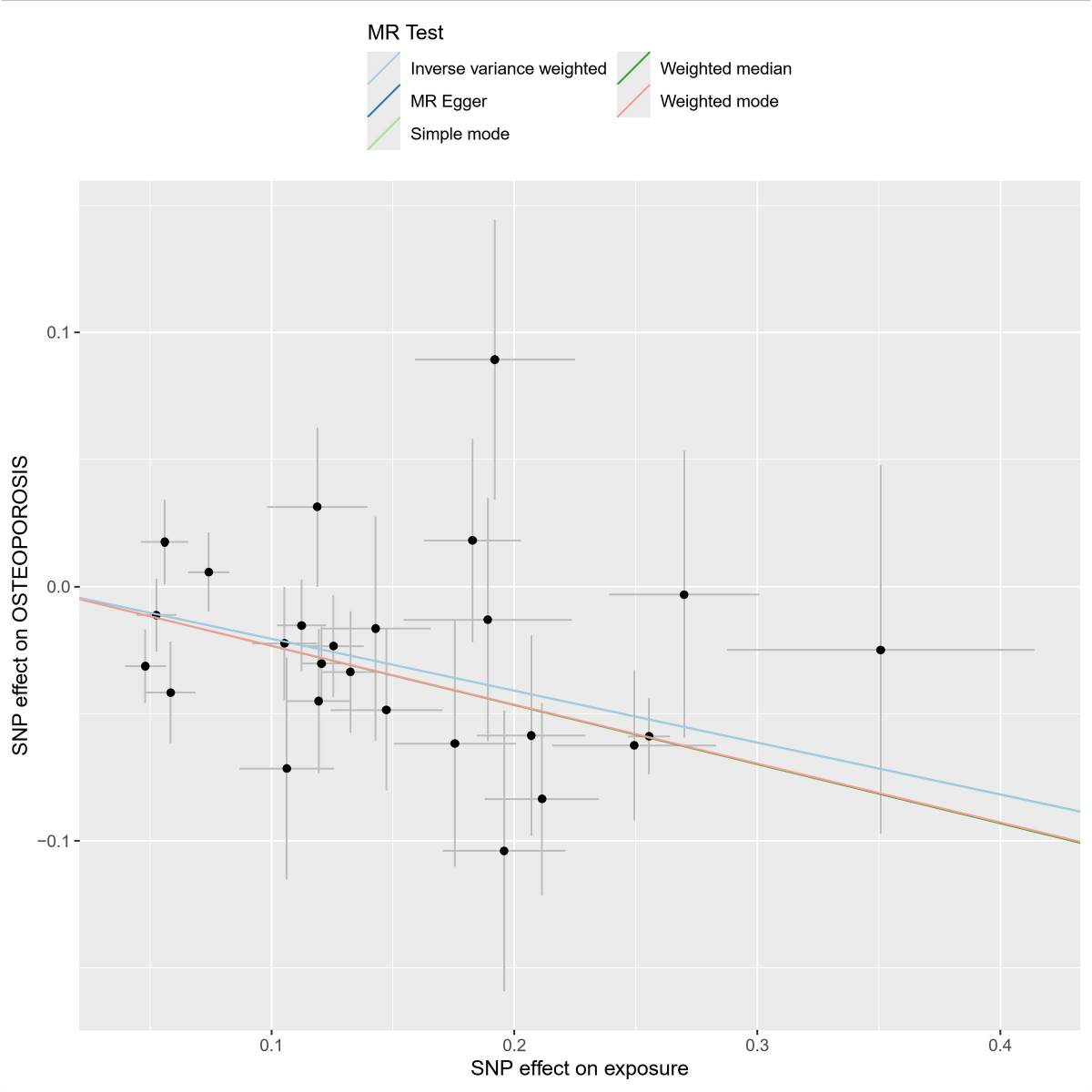

## Slide 4
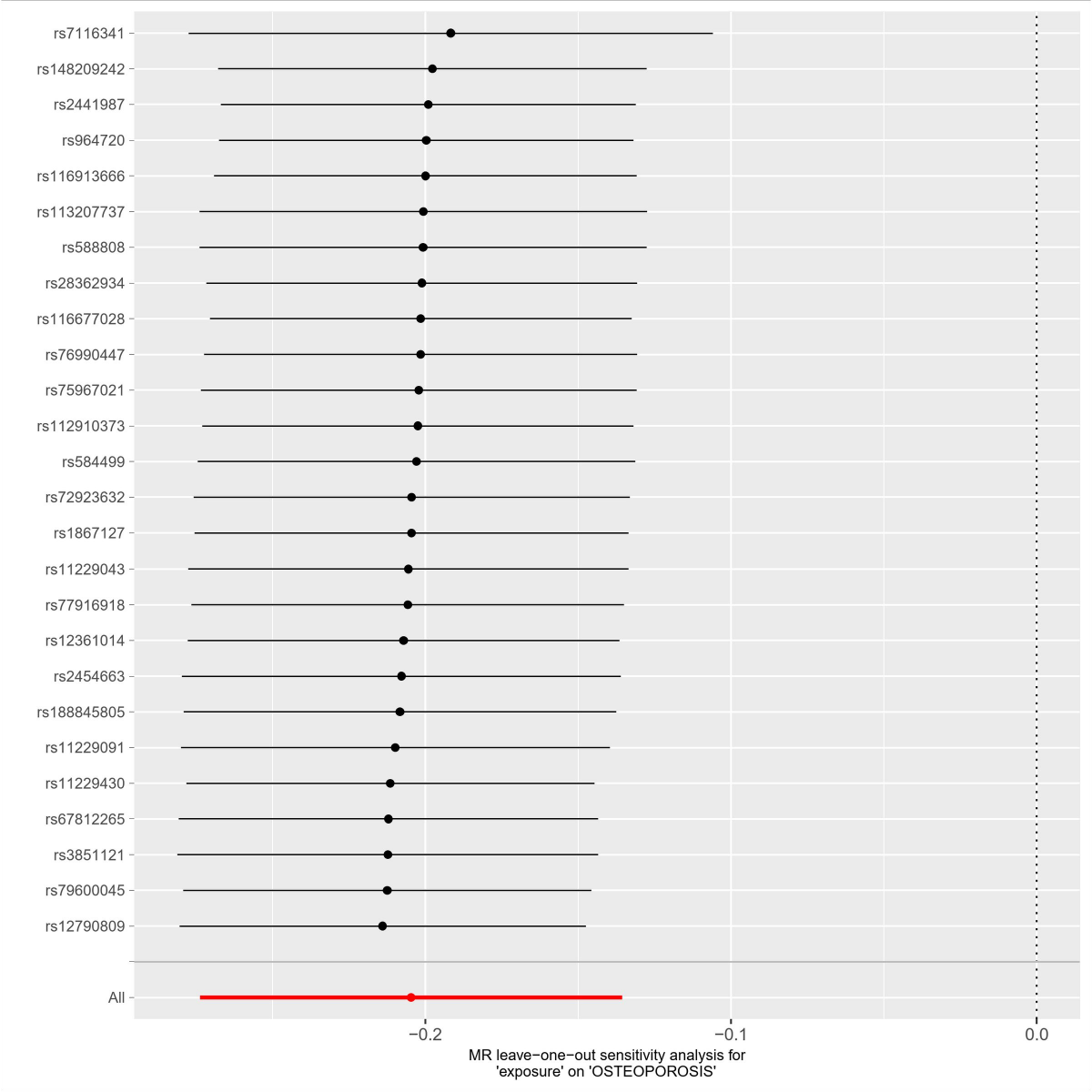

## Slide 5
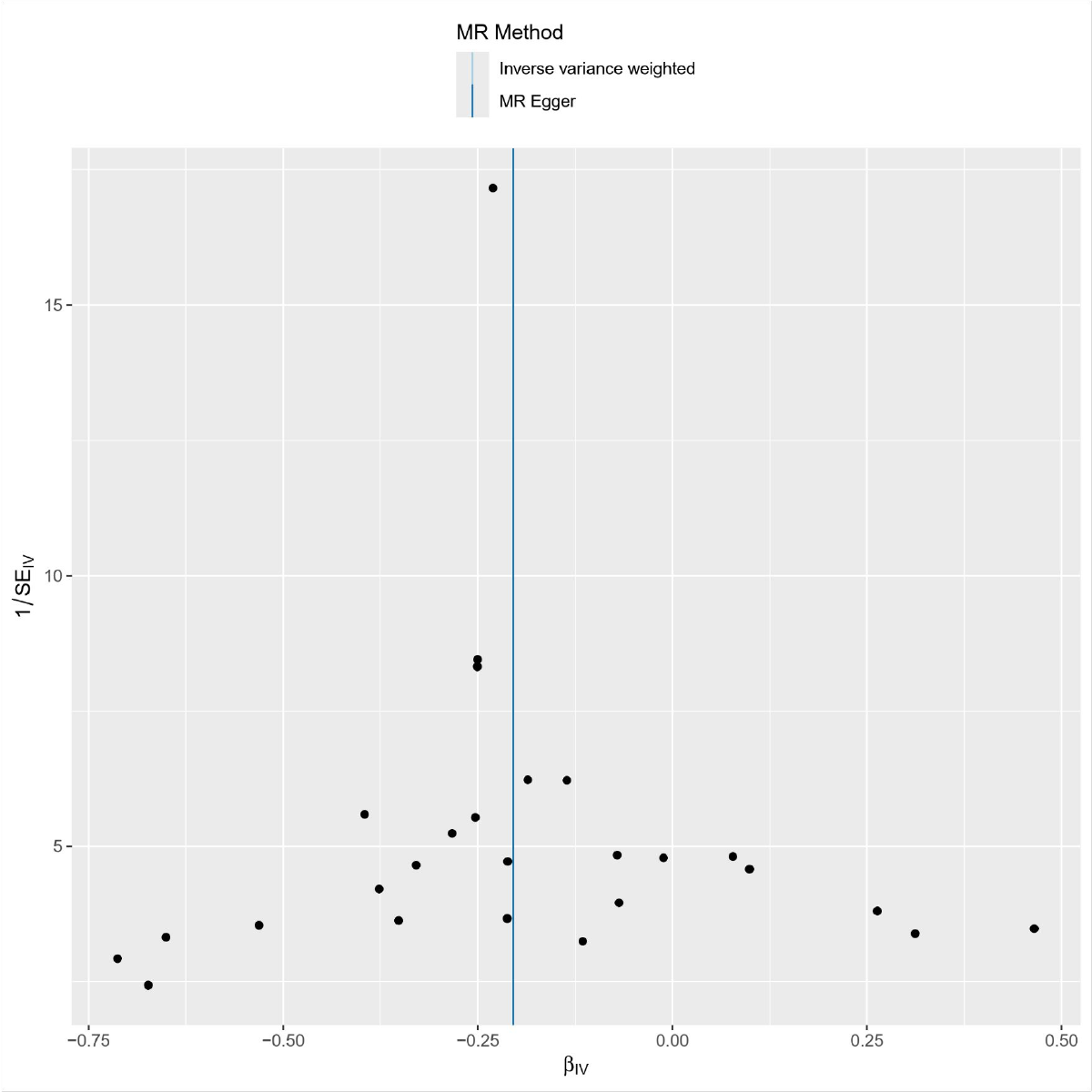

## Slide 6
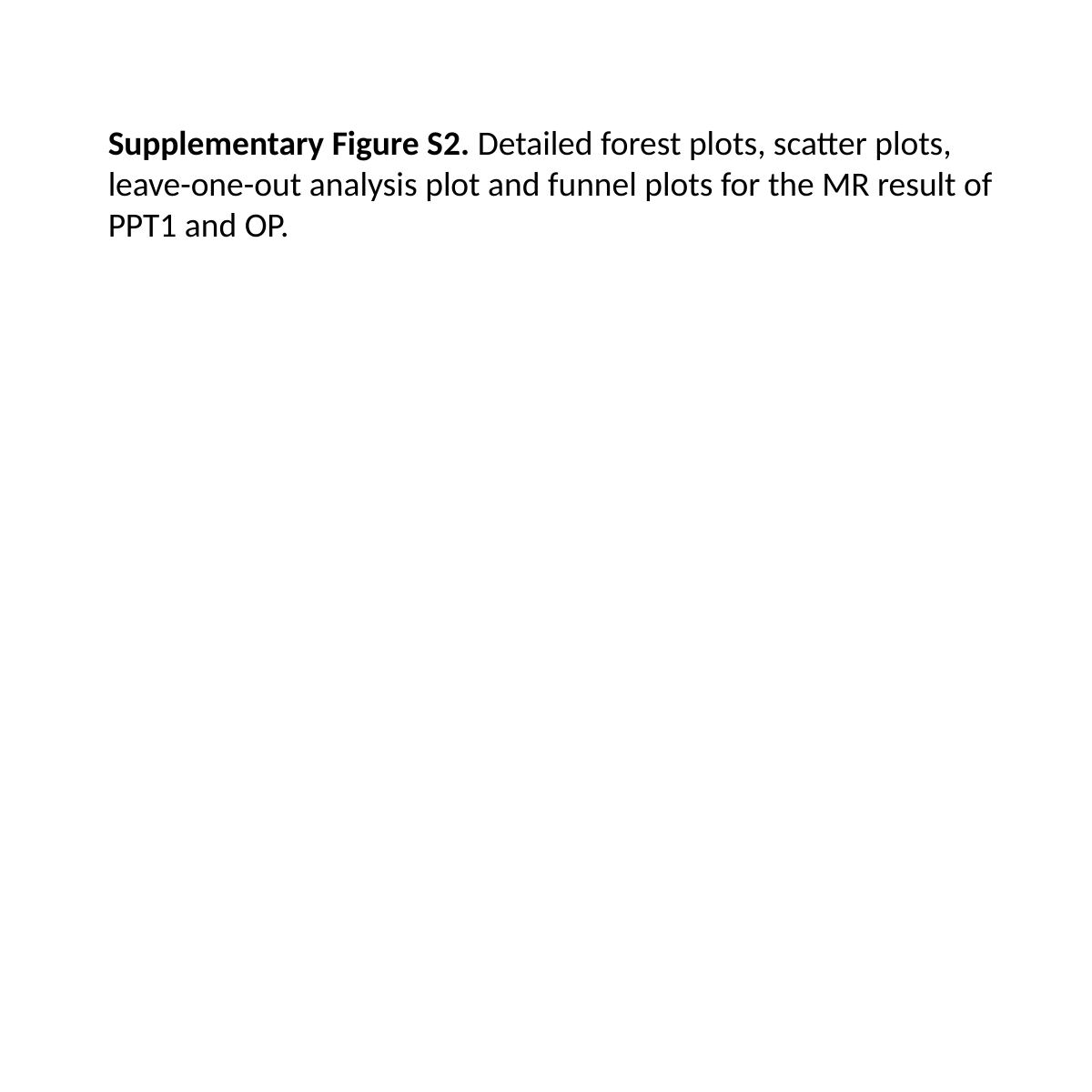

Supplementary Figure S2. Detailed forest plots, scatter plots, leave-one-out analysis plot and funnel plots for the MR result of PPT1 and OP.

## Slide 7
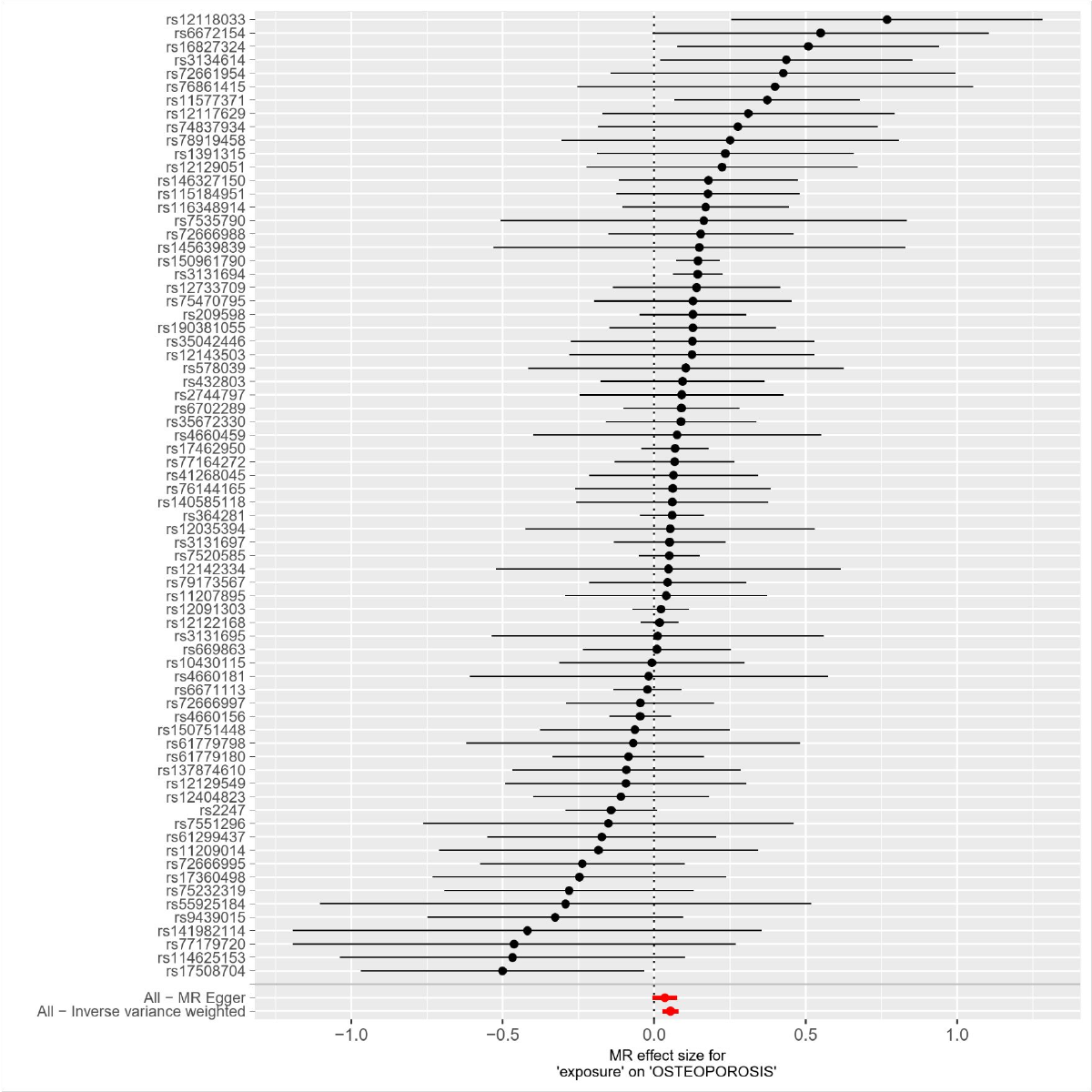

## Slide 8
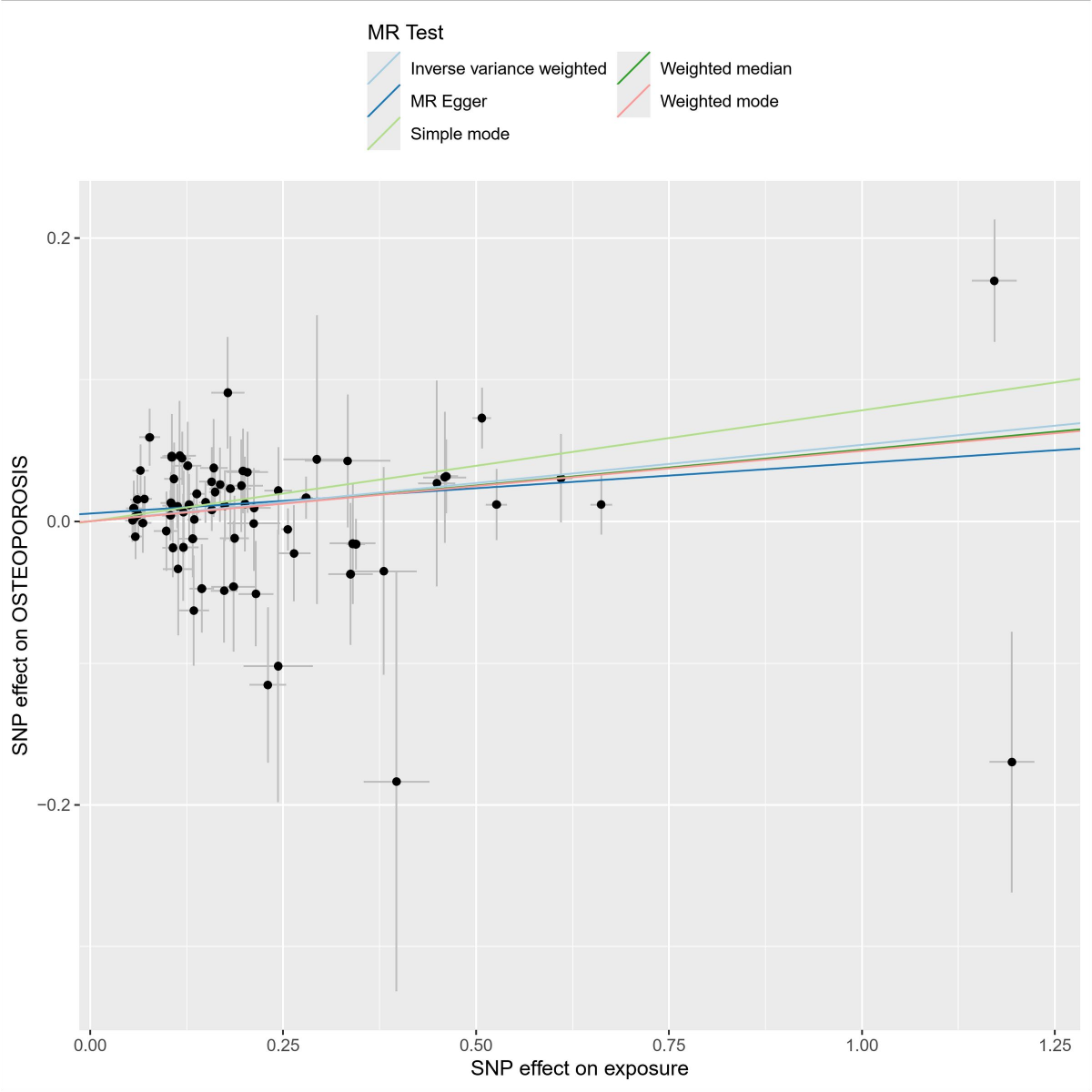

## Slide 9
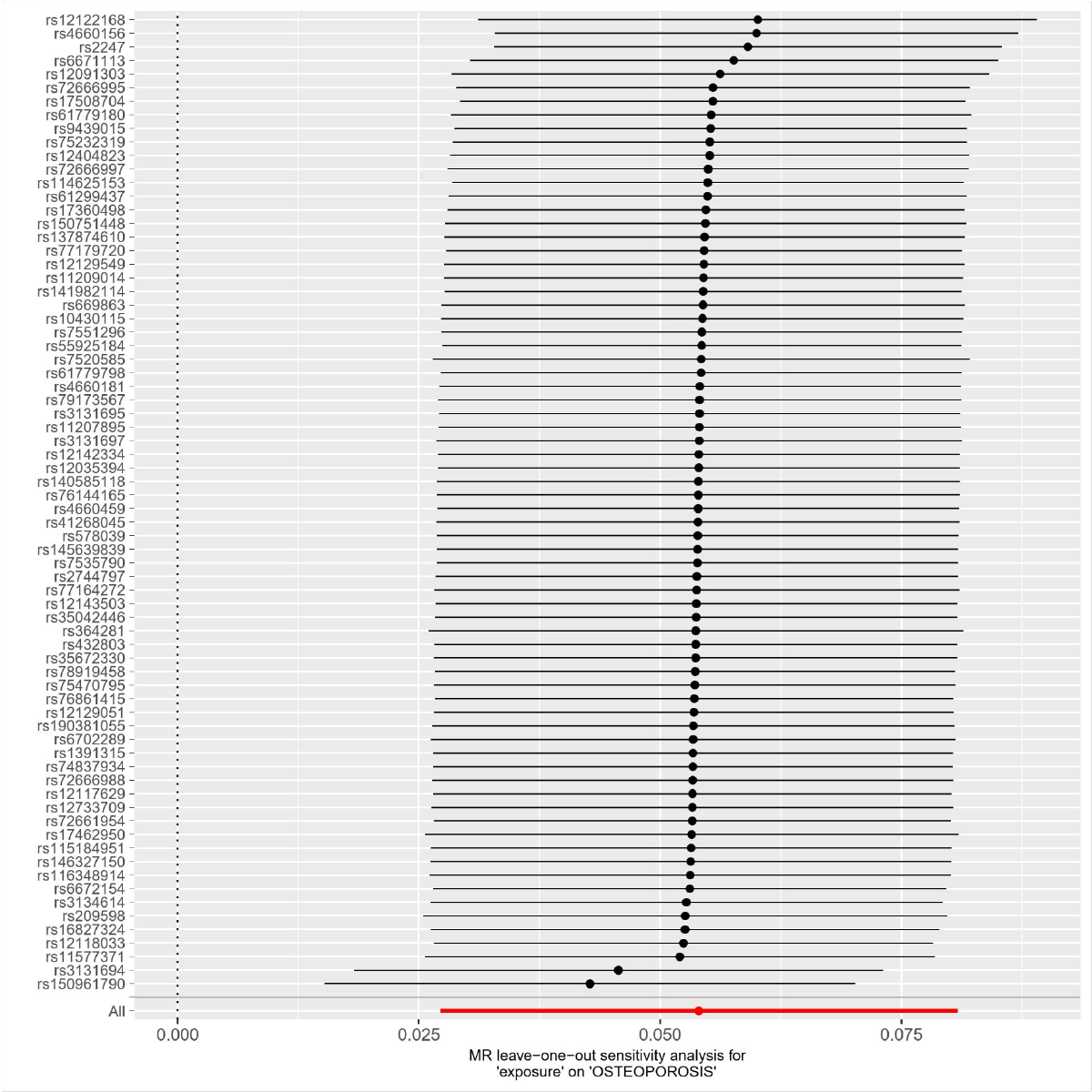

## Slide 10
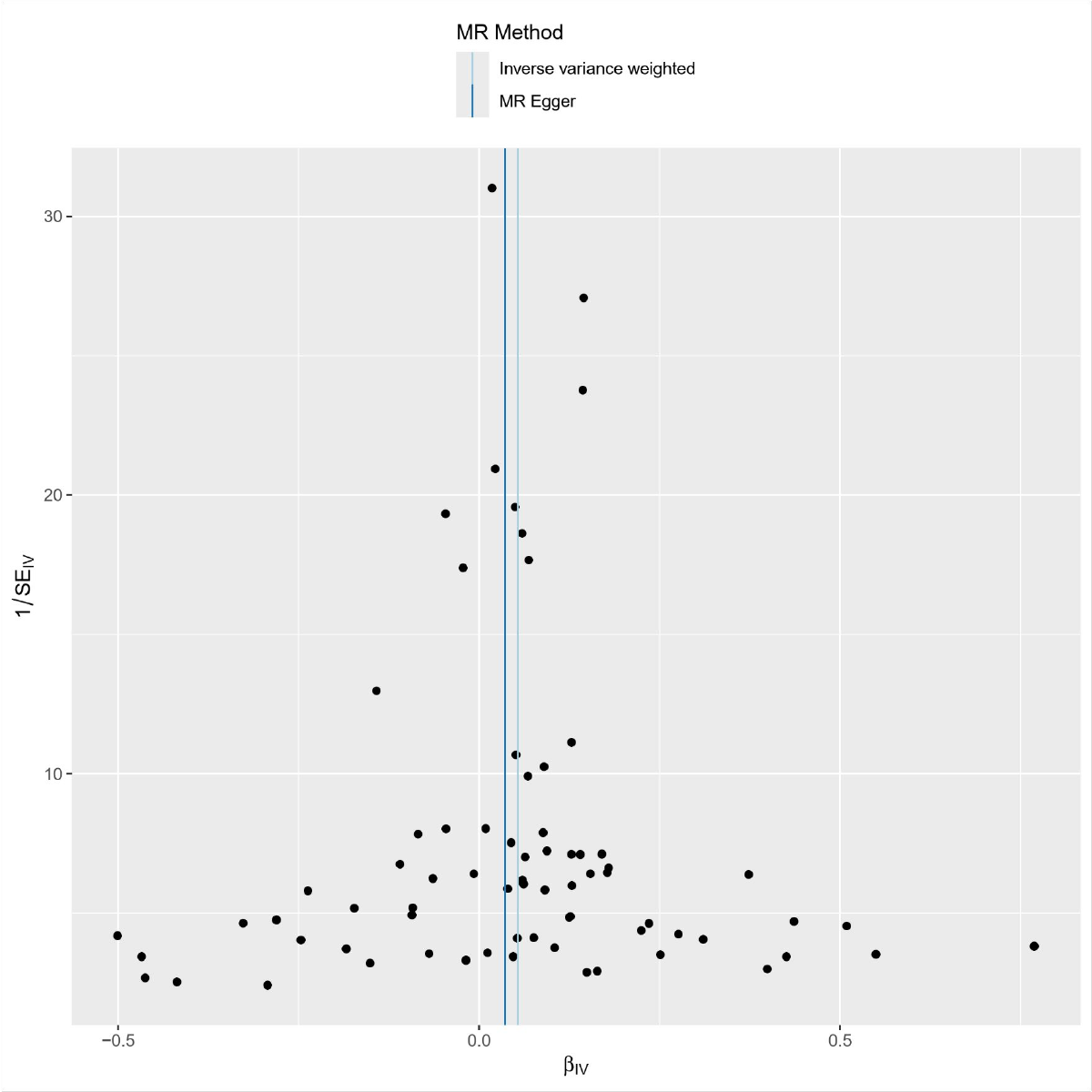

## Slide 11
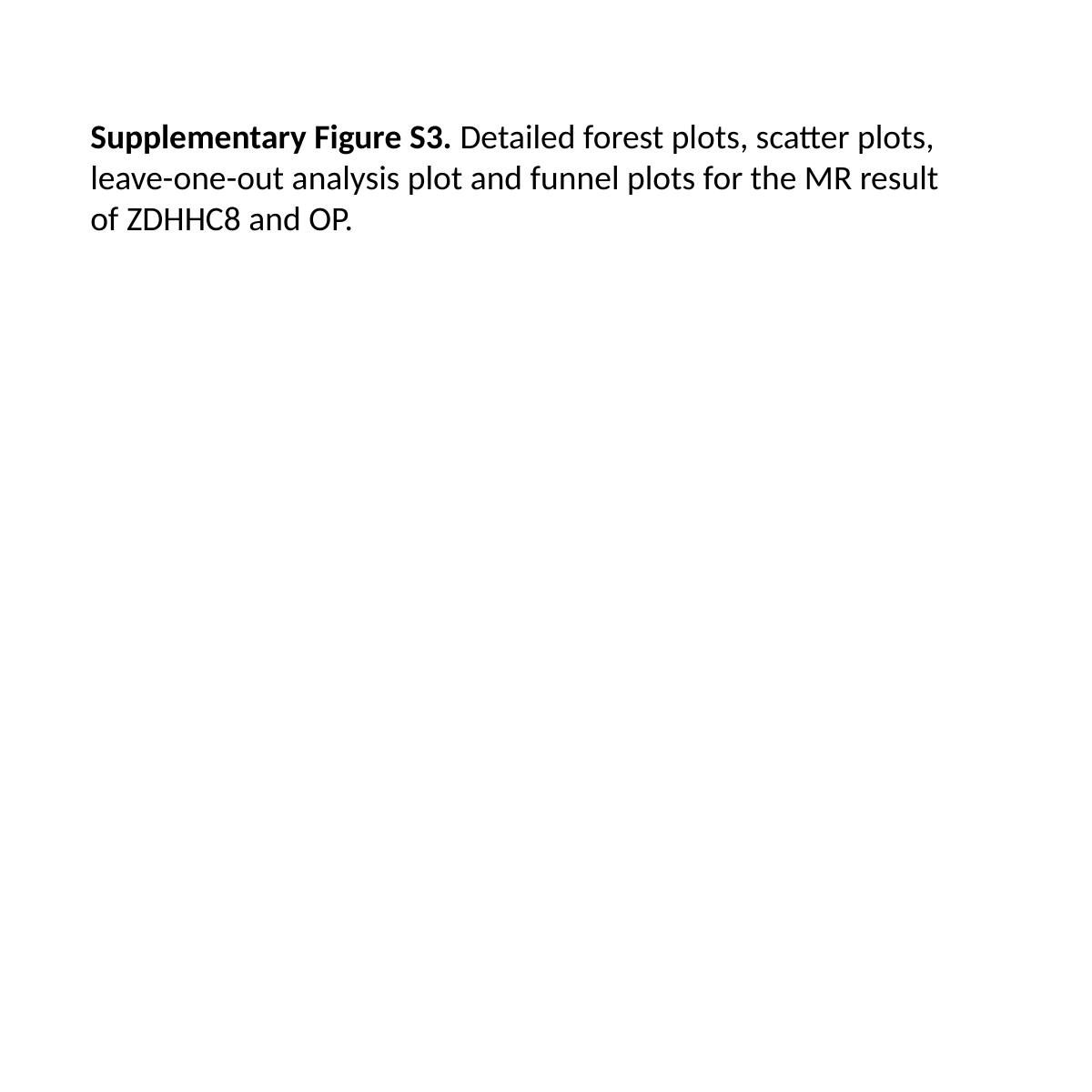

Supplementary Figure S3. Detailed forest plots, scatter plots, leave-one-out analysis plot and funnel plots for the MR result of ZDHHC8 and OP.

## Slide 12
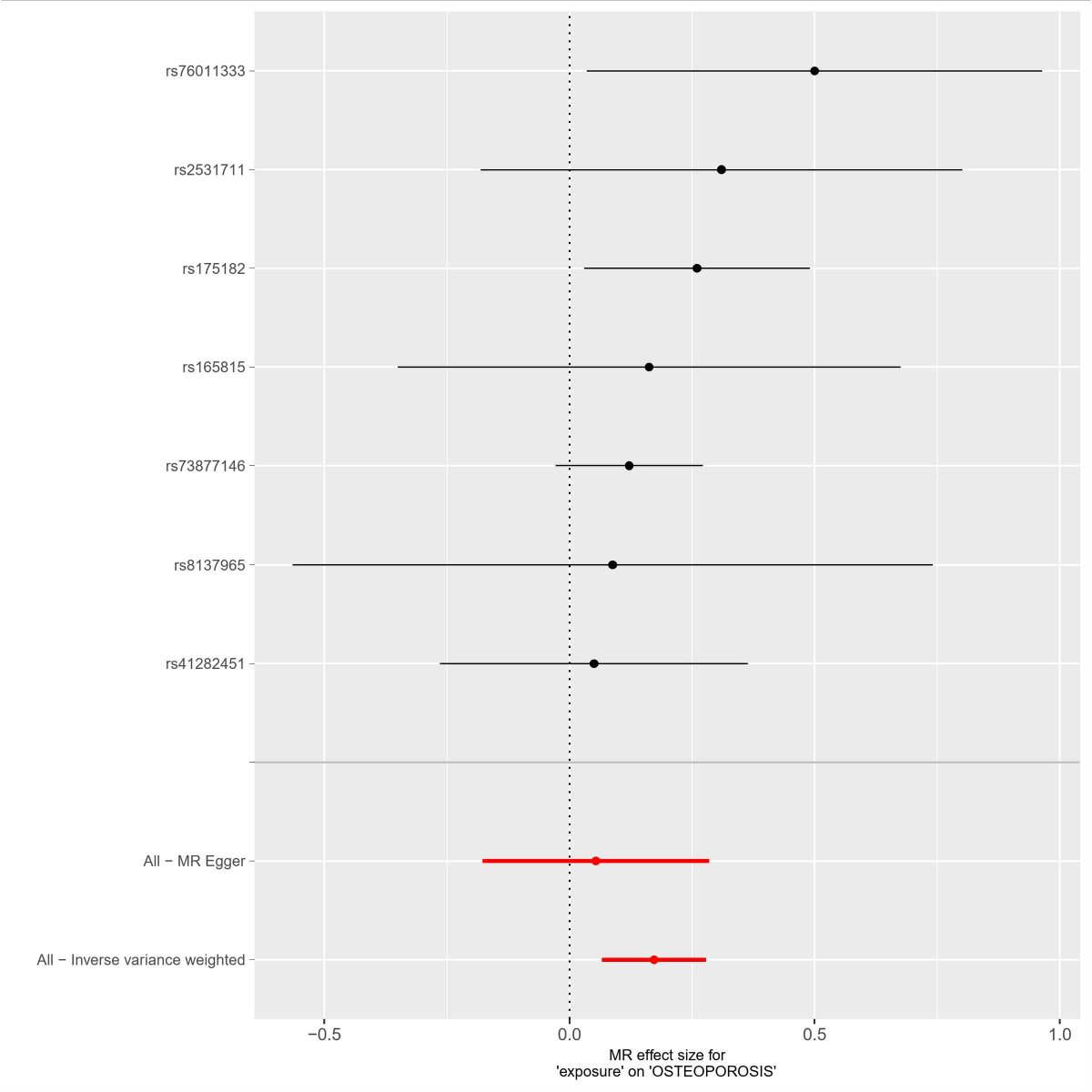

## Slide 13
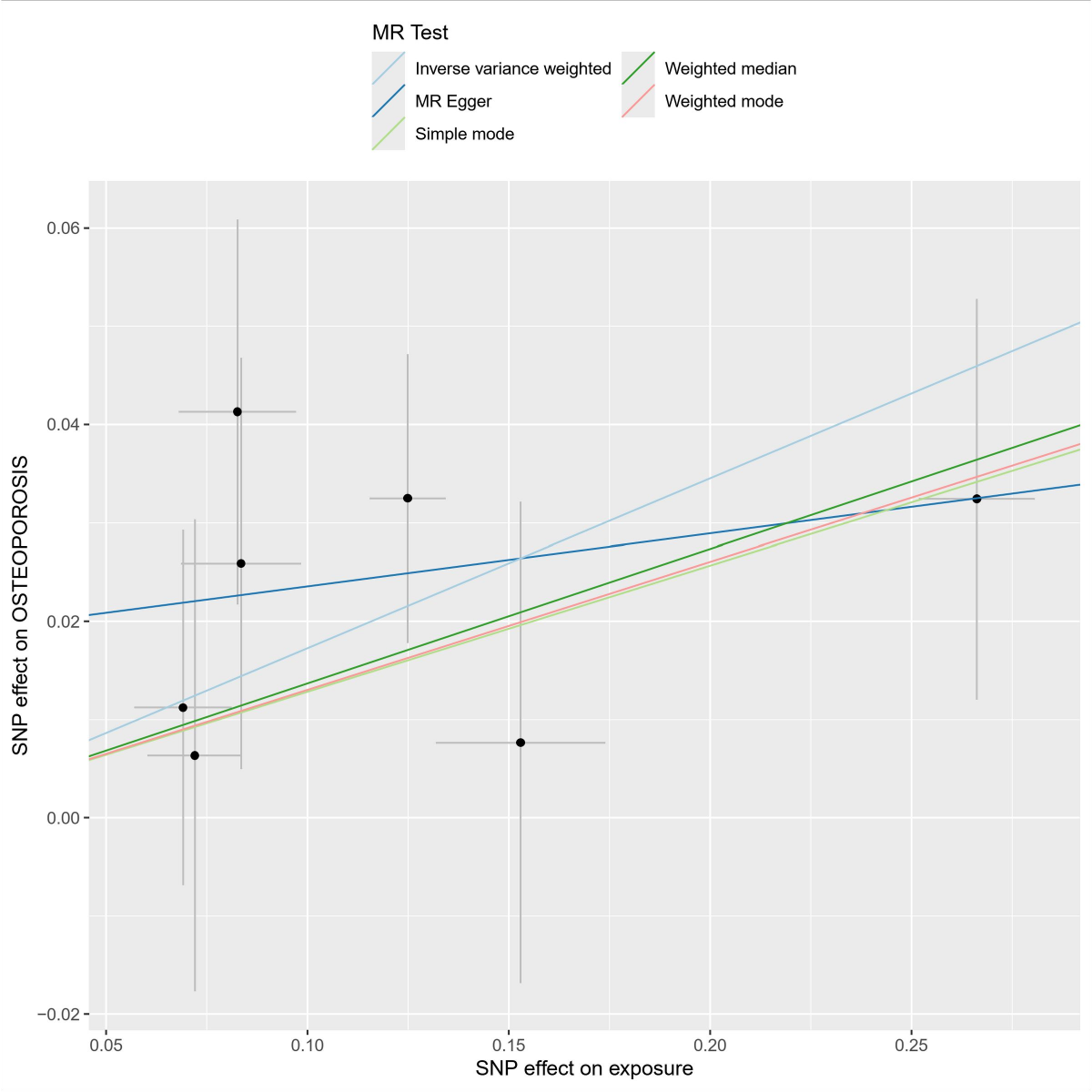

## Slide 14
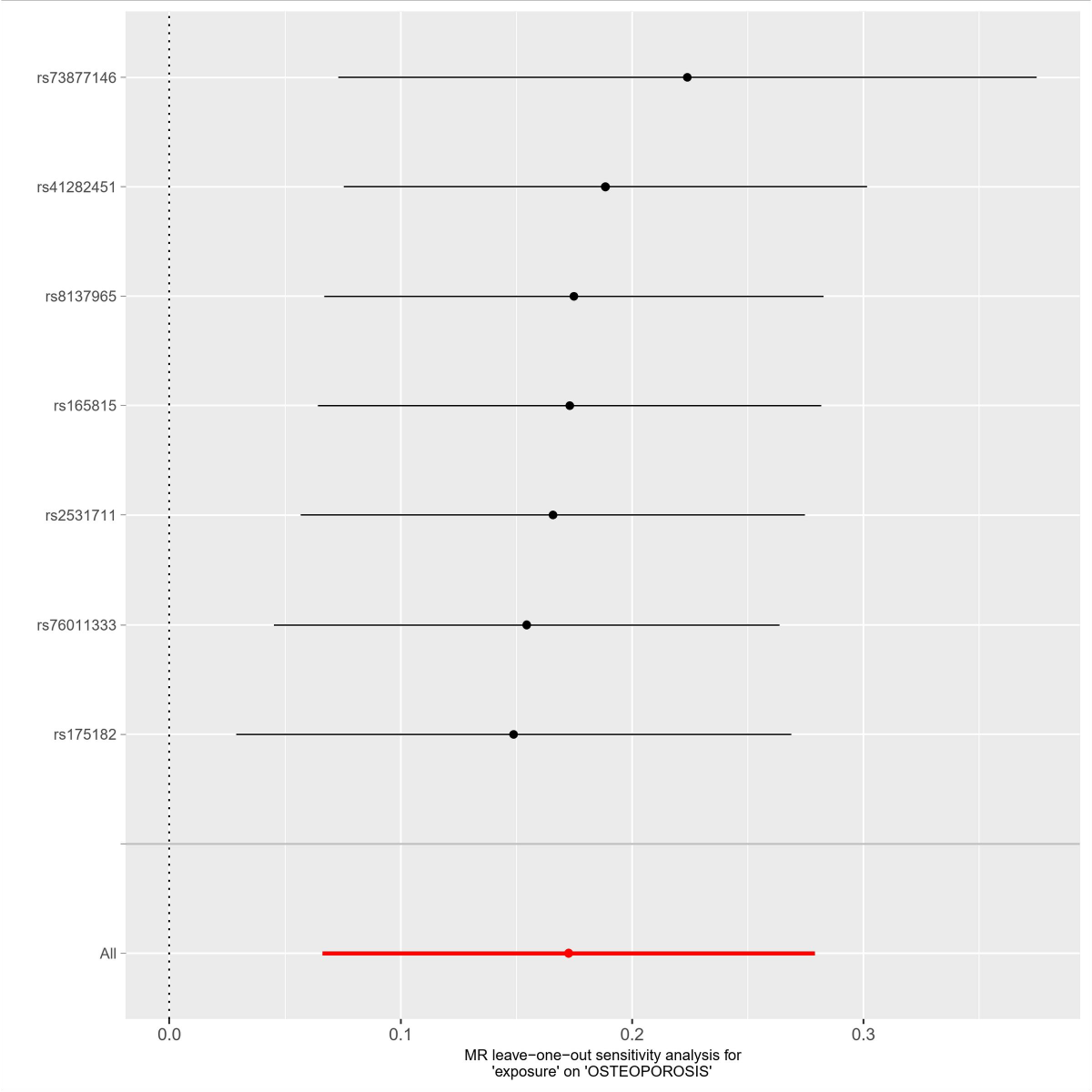

## Slide 15
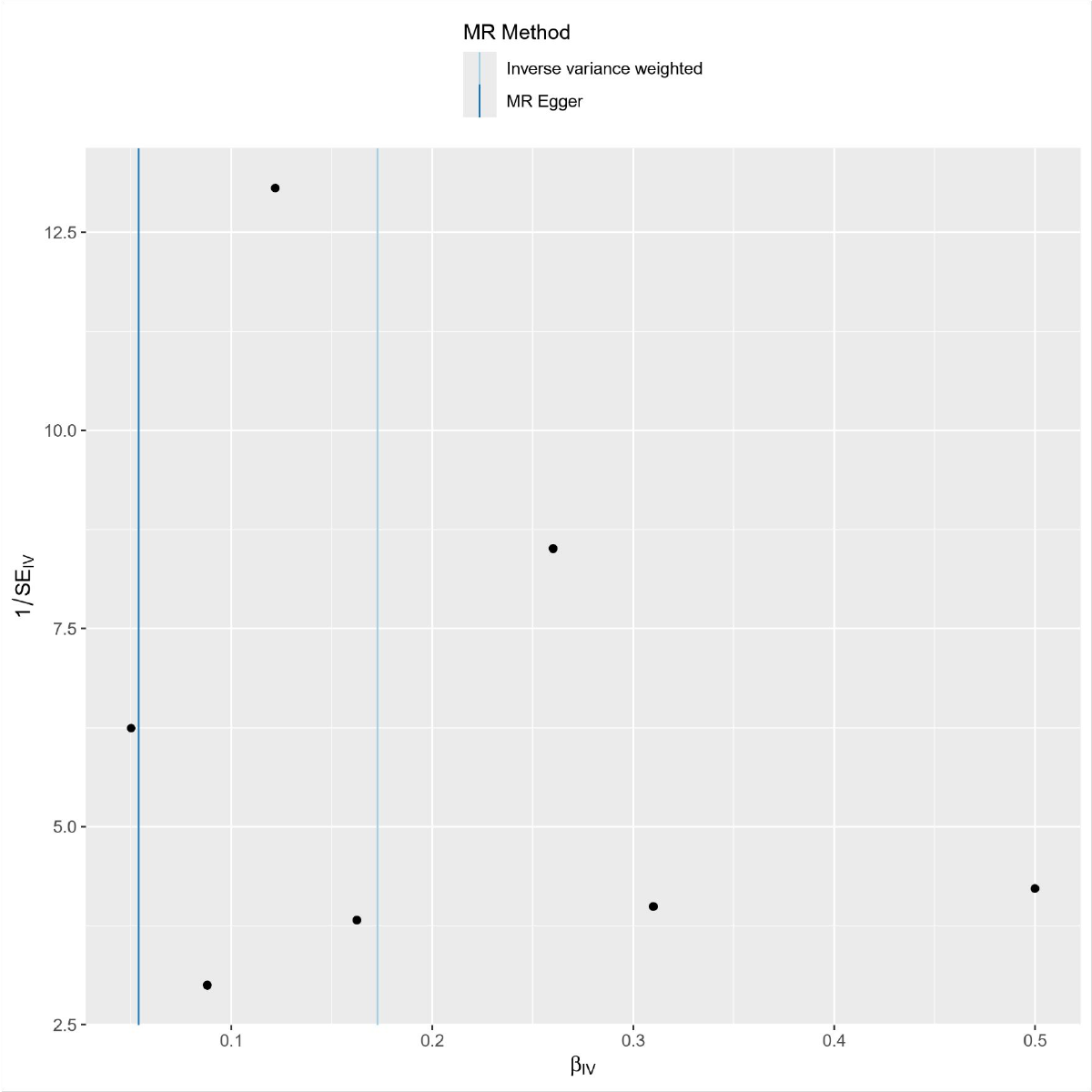

## Slide 16
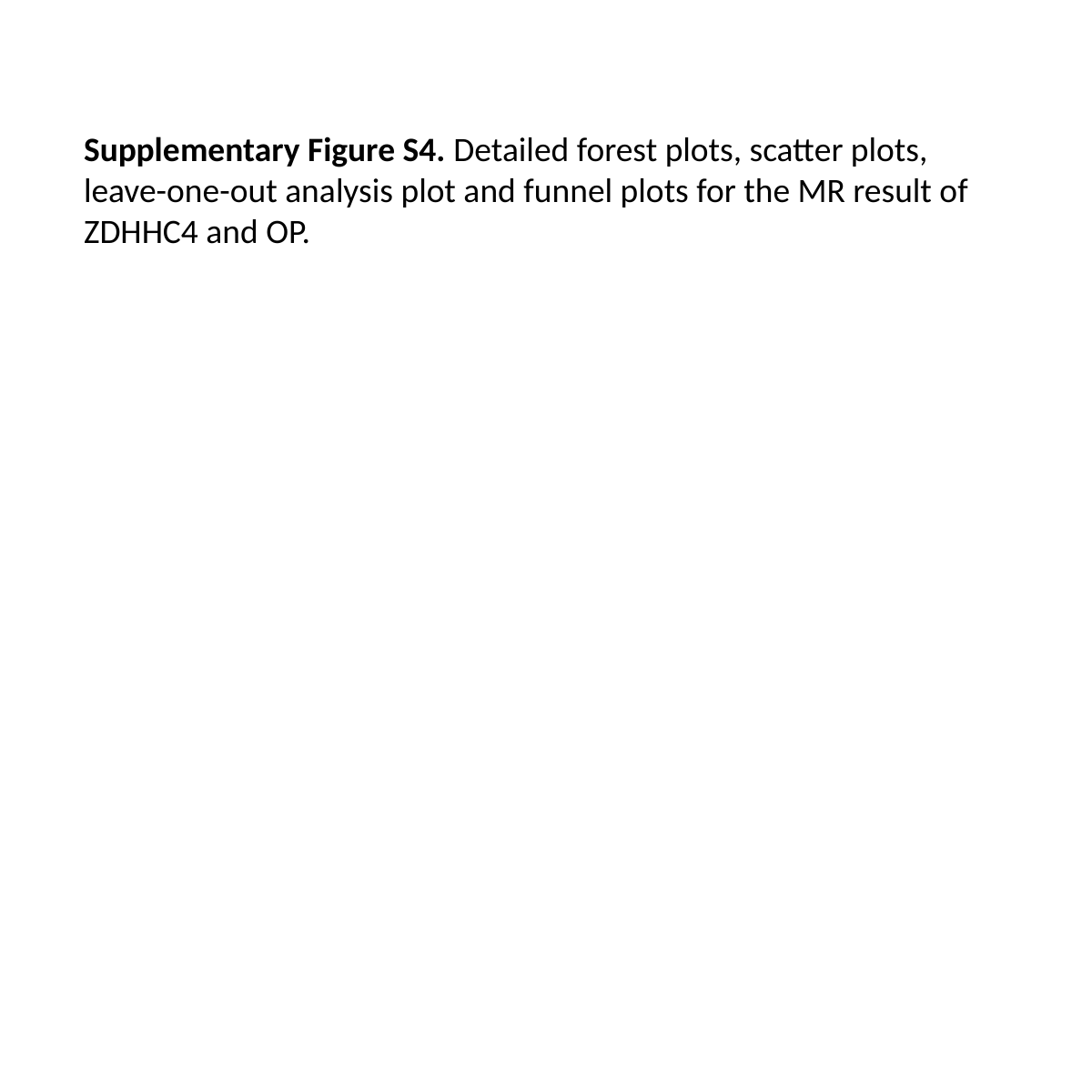

Supplementary Figure S4. Detailed forest plots, scatter plots, leave-one-out analysis plot and funnel plots for the MR result of ZDHHC4 and OP.

## Slide 17
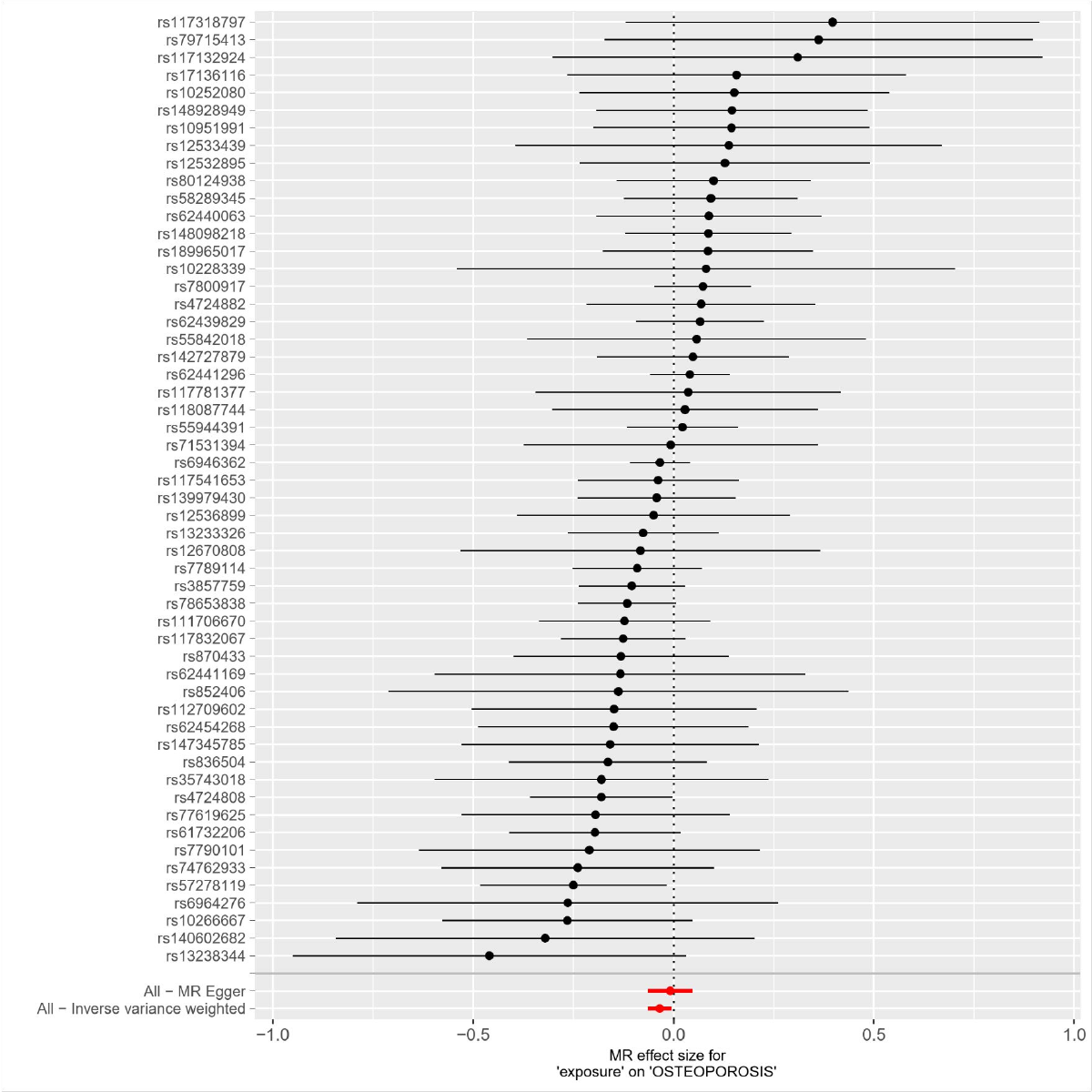

## Slide 18
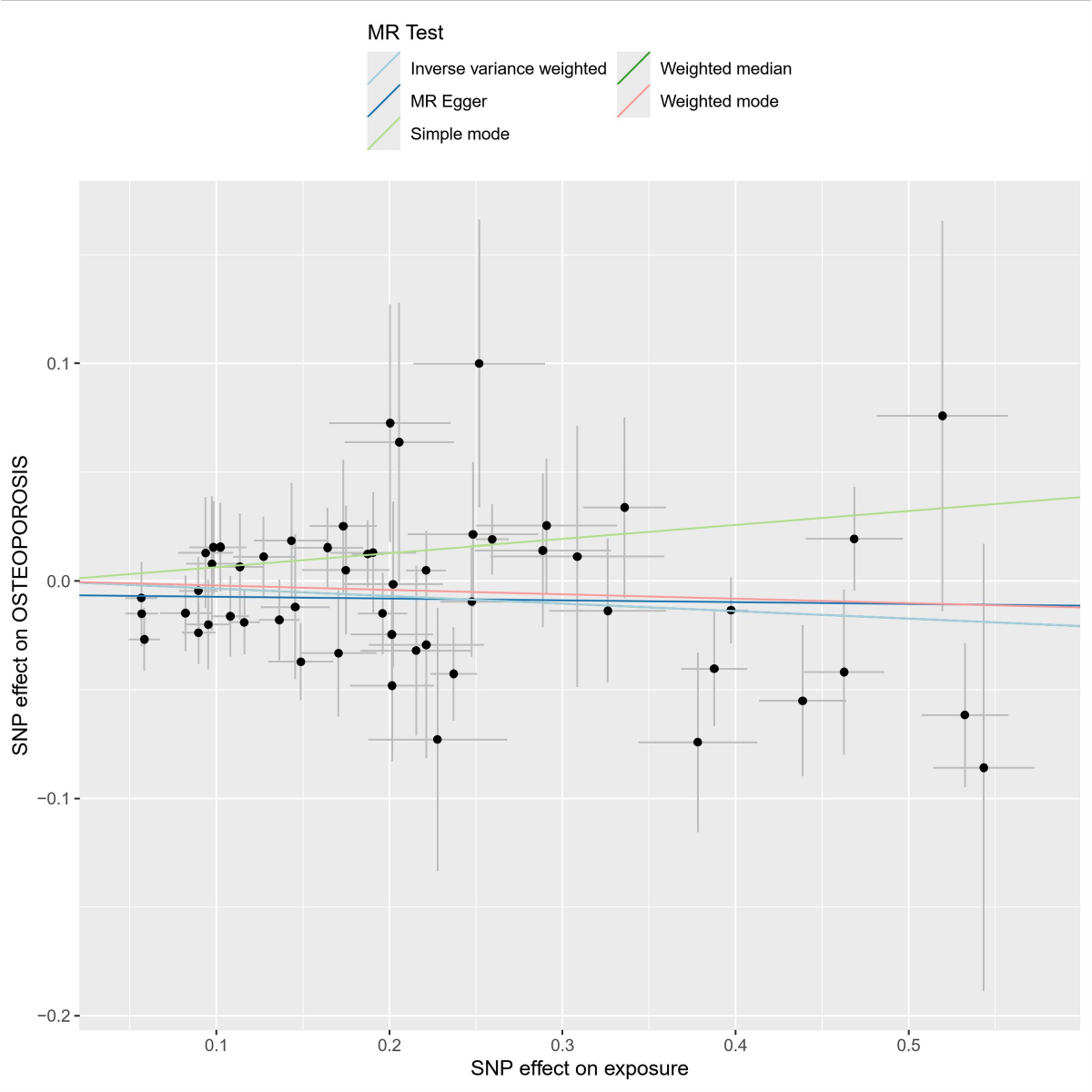

## Slide 19
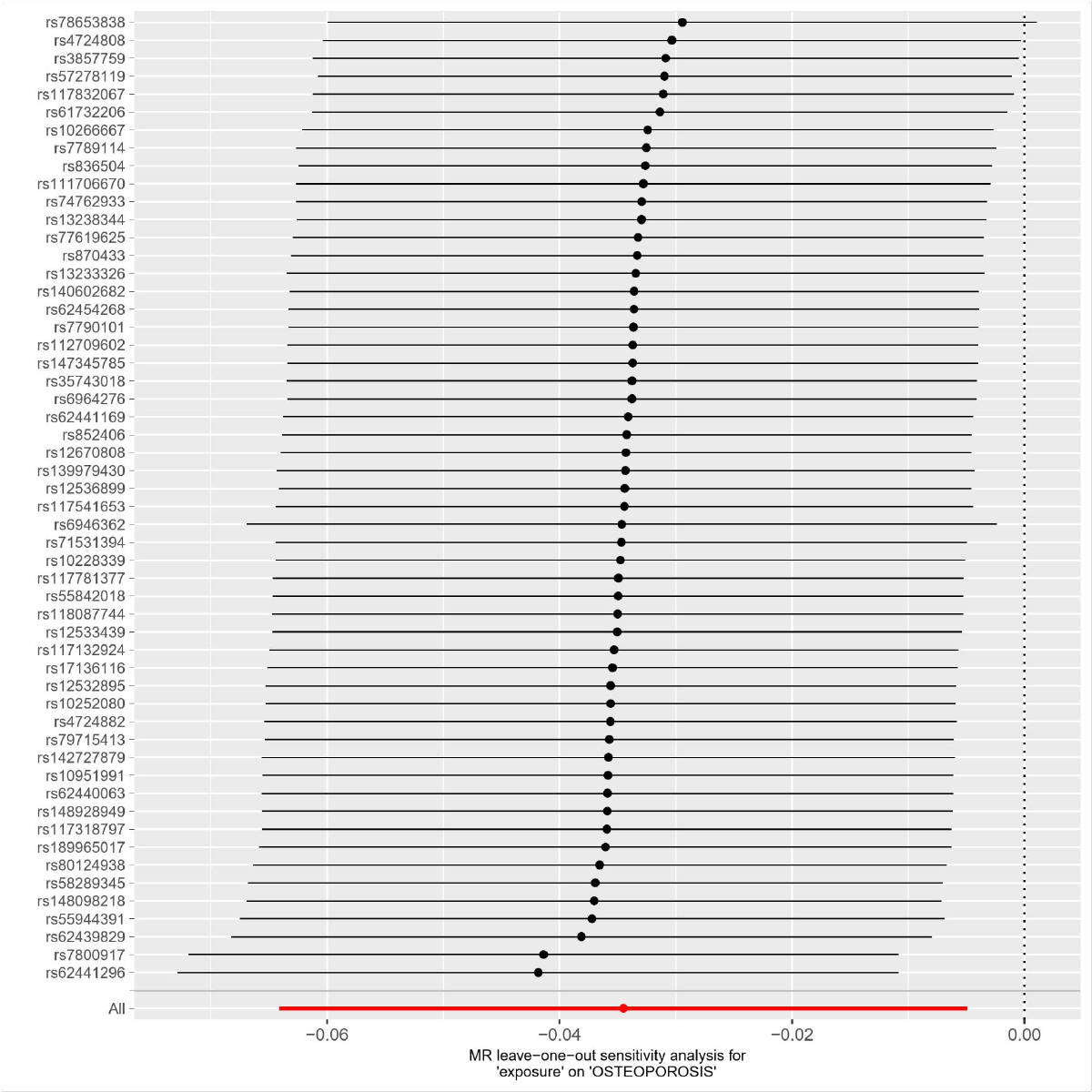

## Slide 20
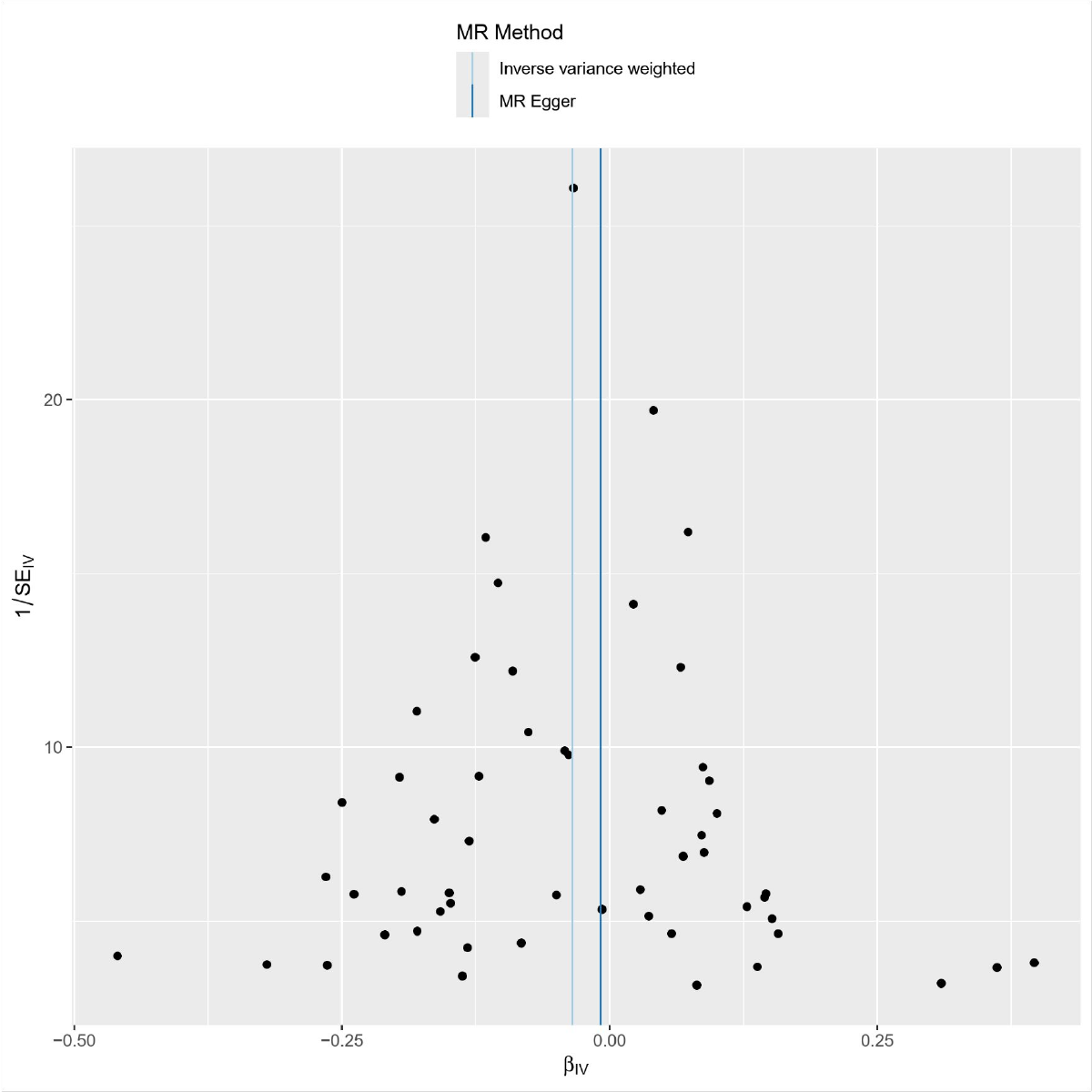

## Slide 21
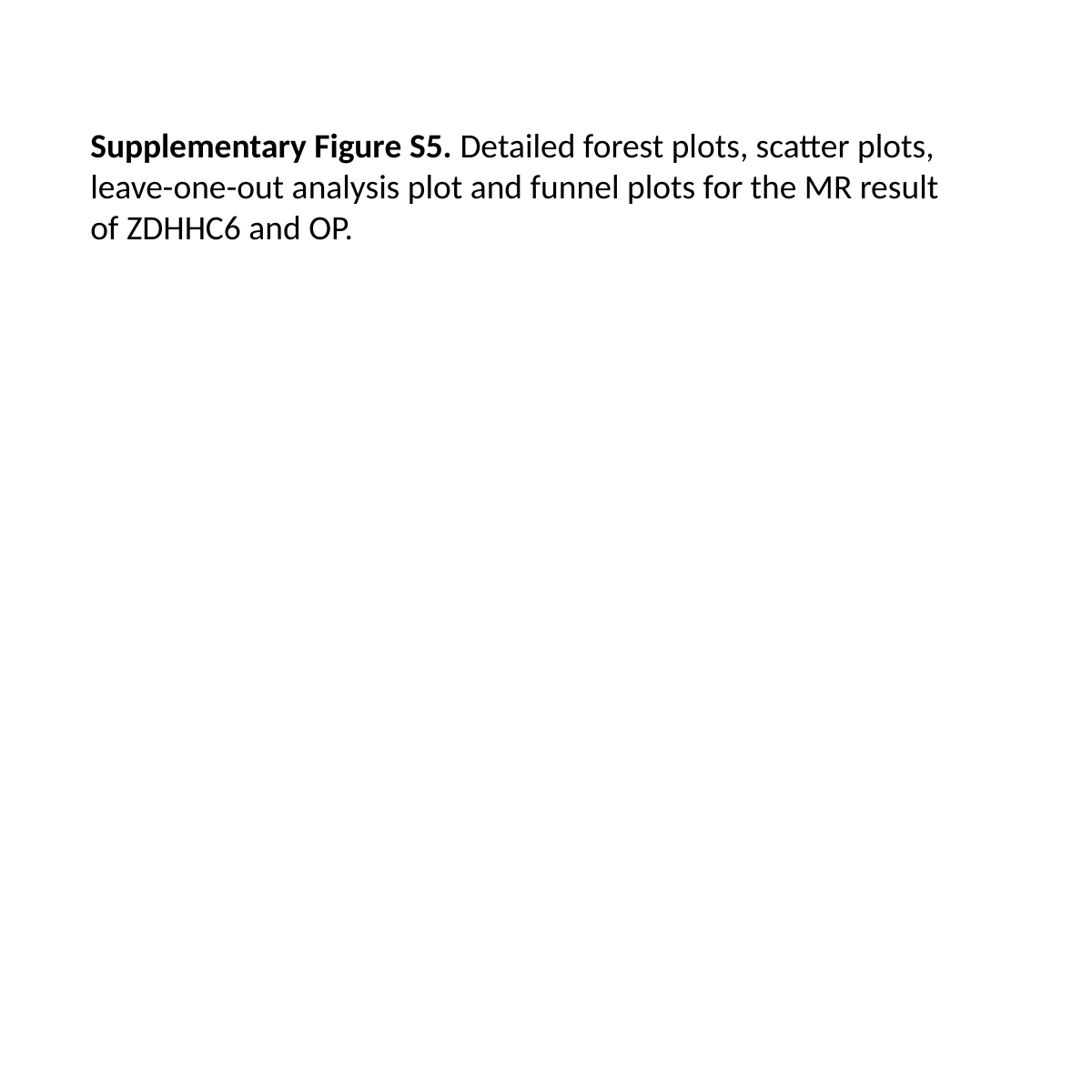

Supplementary Figure S5. Detailed forest plots, scatter plots, leave-one-out analysis plot and funnel plots for the MR result of ZDHHC6 and OP.

## Slide 22
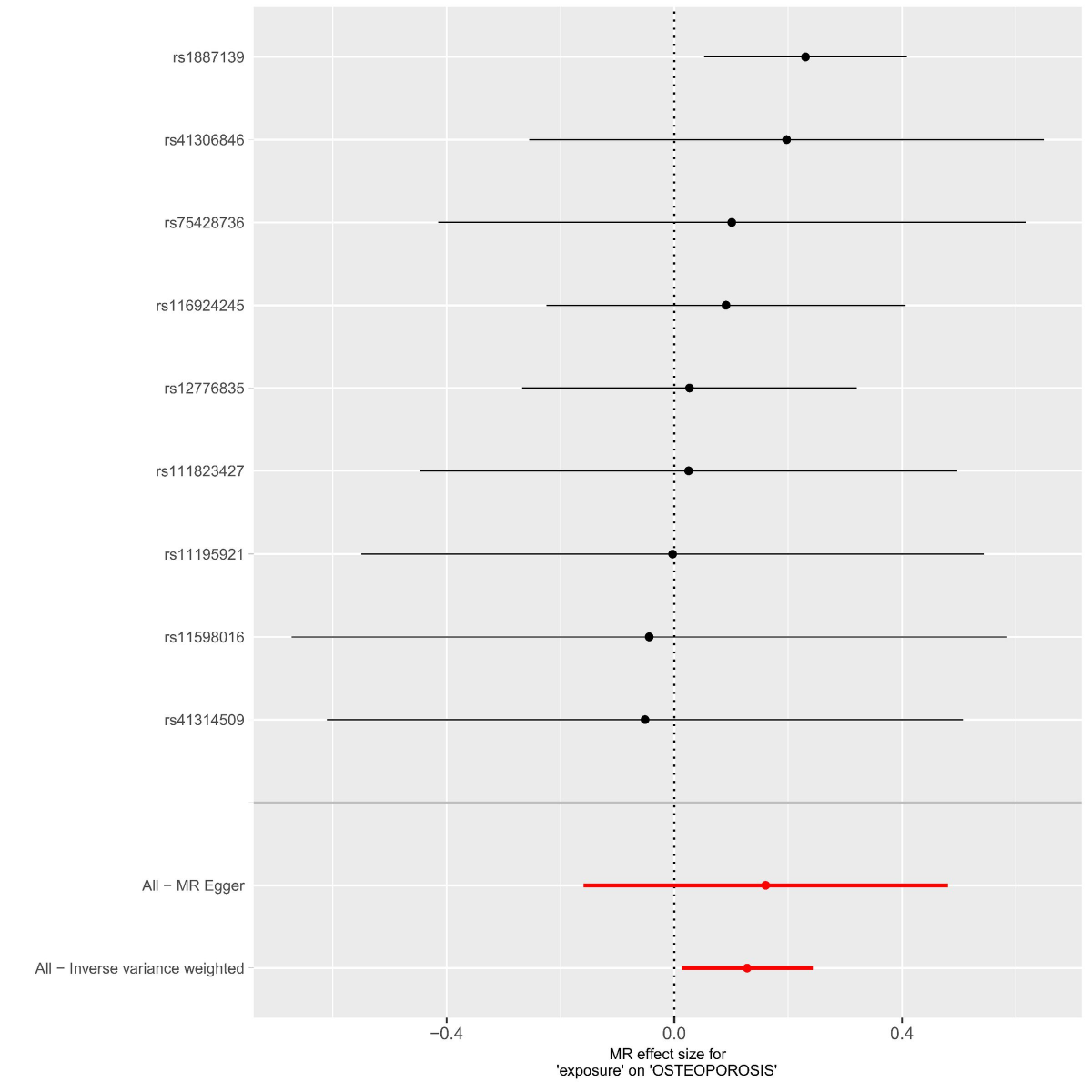

## Slide 23
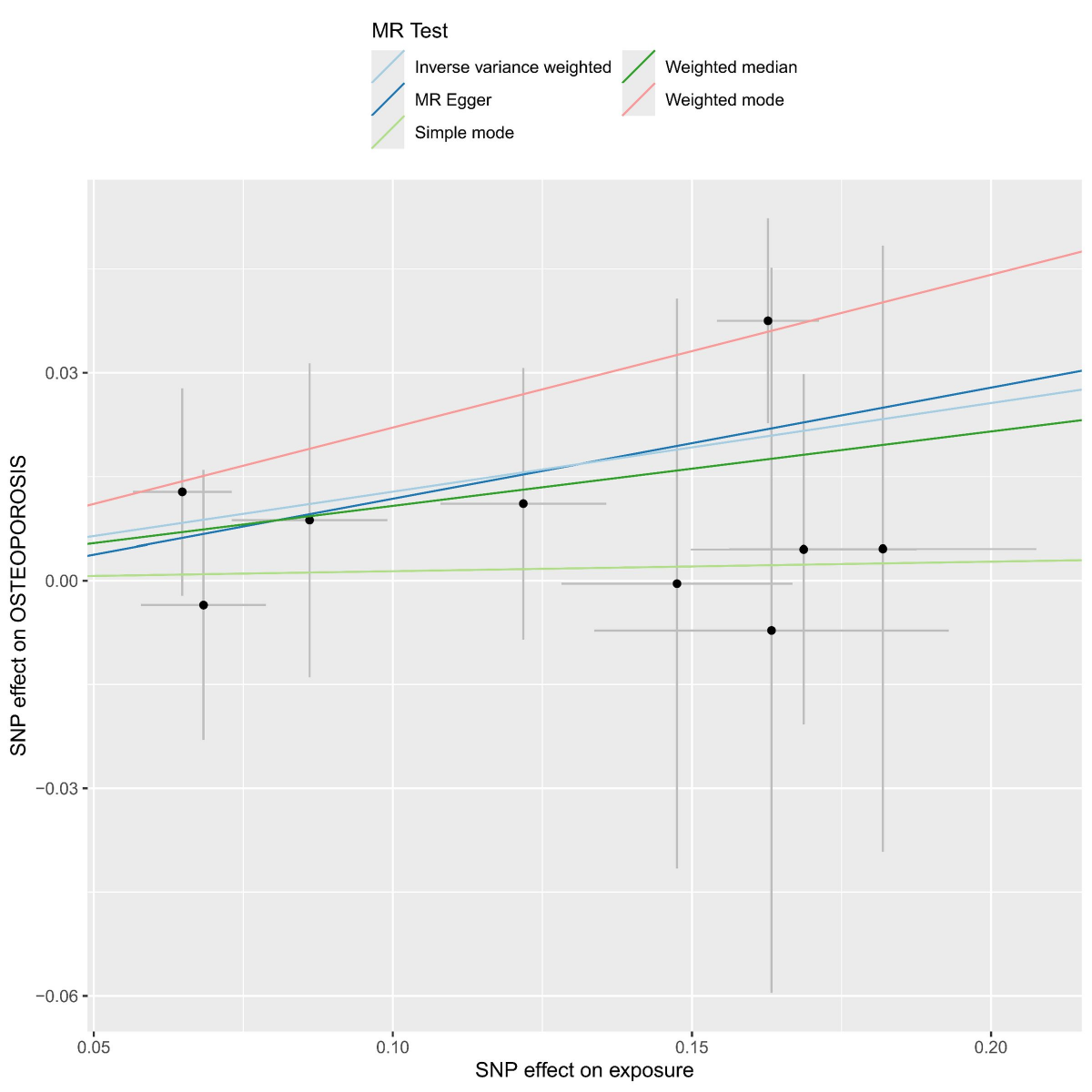

## Slide 24
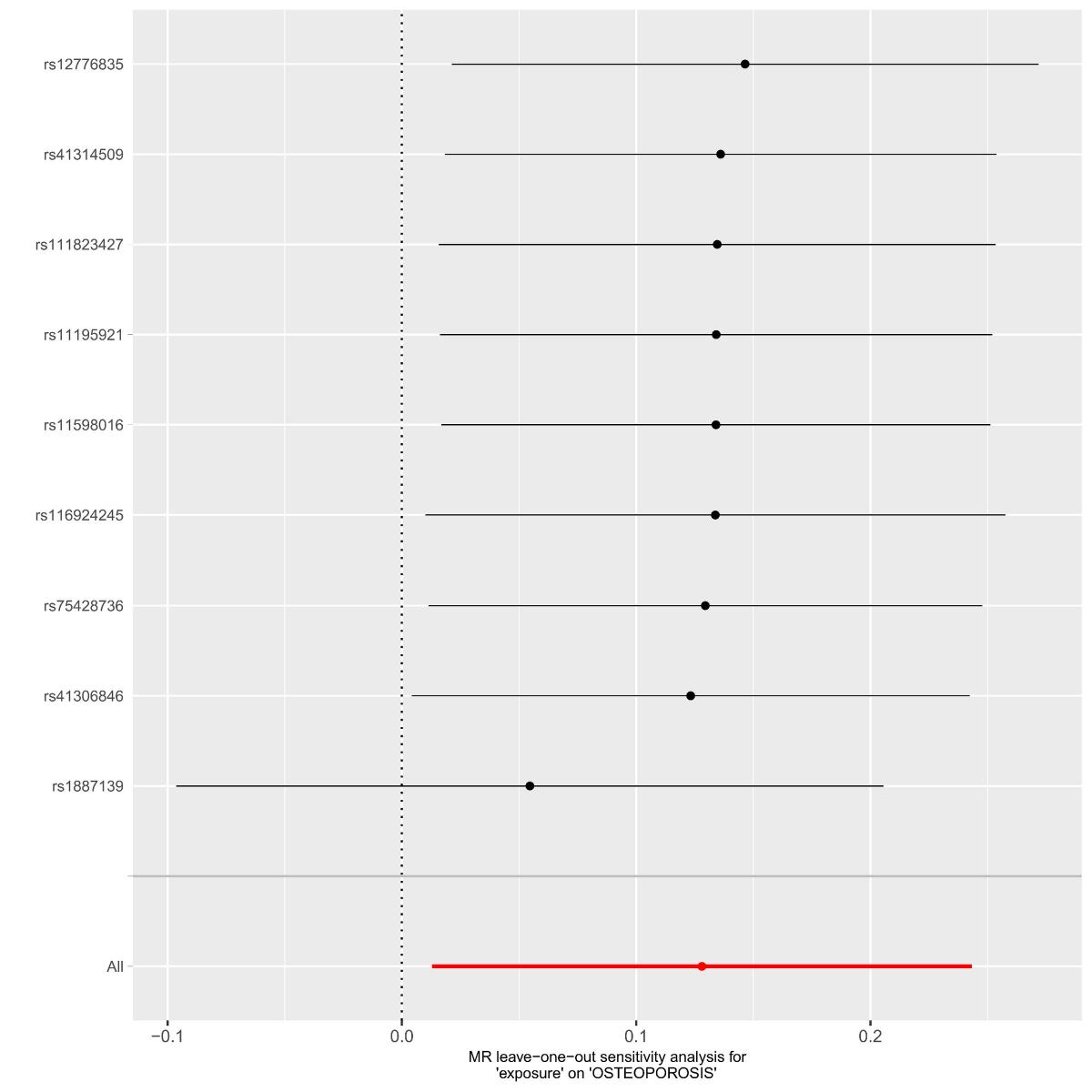

## Slide 25
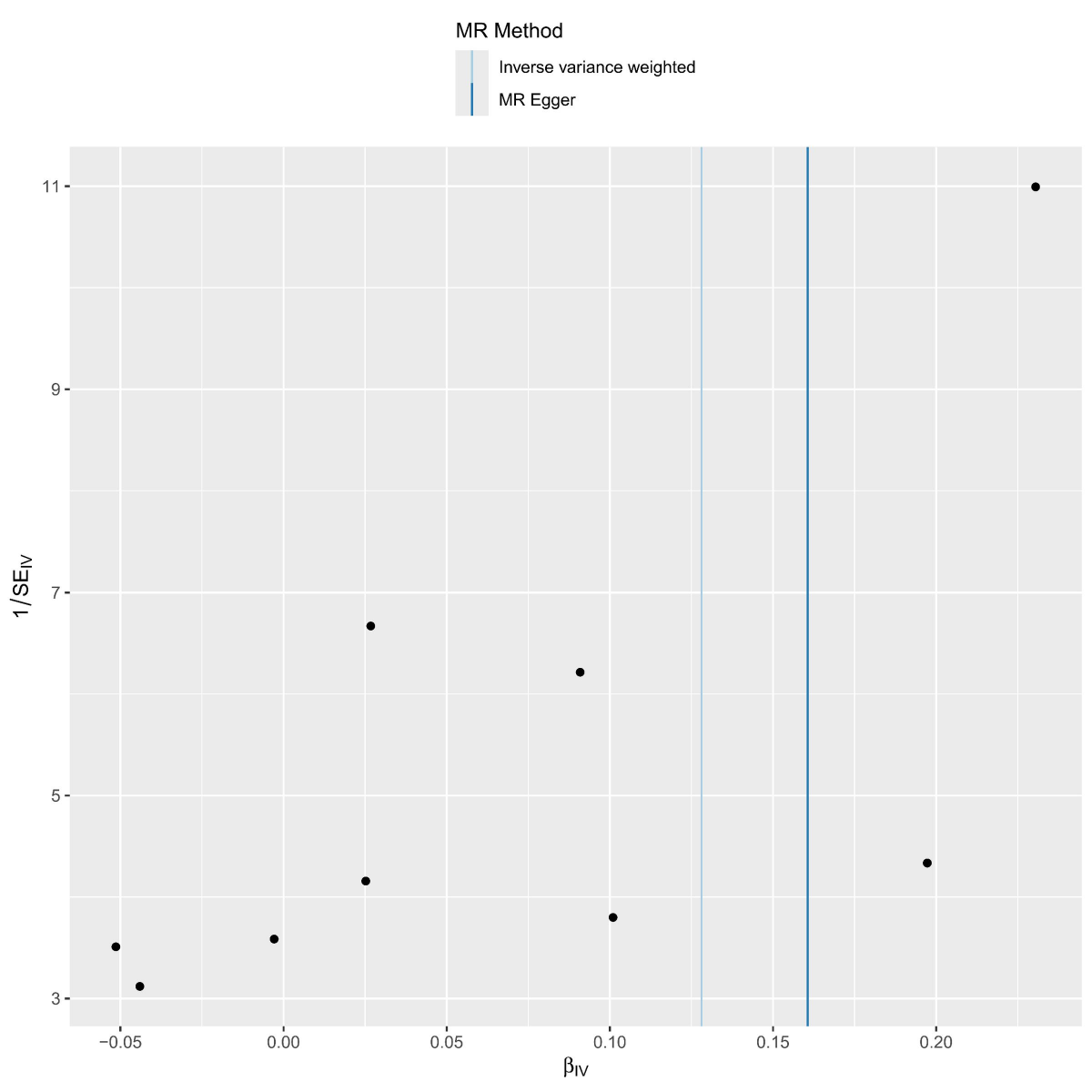

## Slide 26
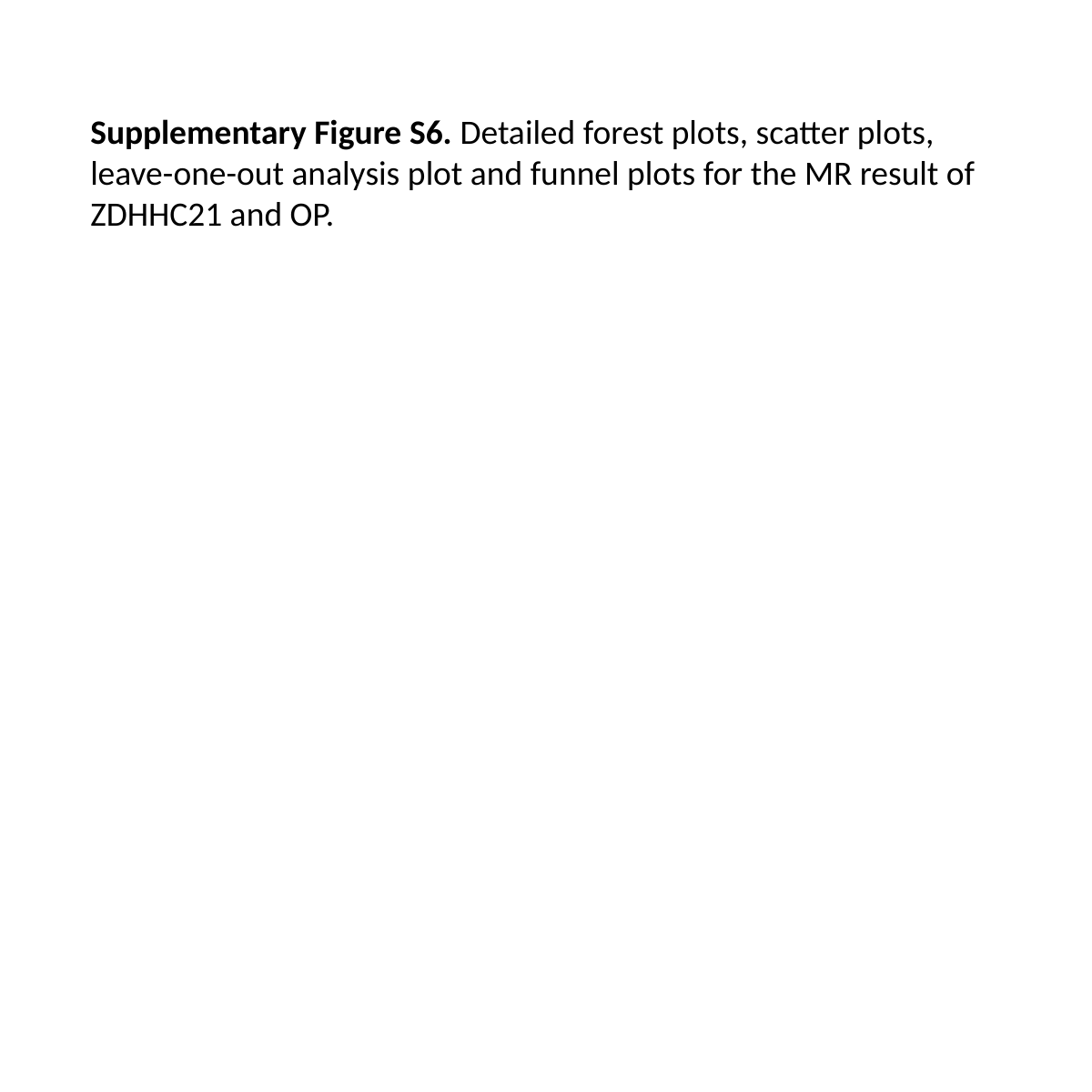

Supplementary Figure S6. Detailed forest plots, scatter plots, leave-one-out analysis plot and funnel plots for the MR result of ZDHHC21 and OP.

## Slide 27
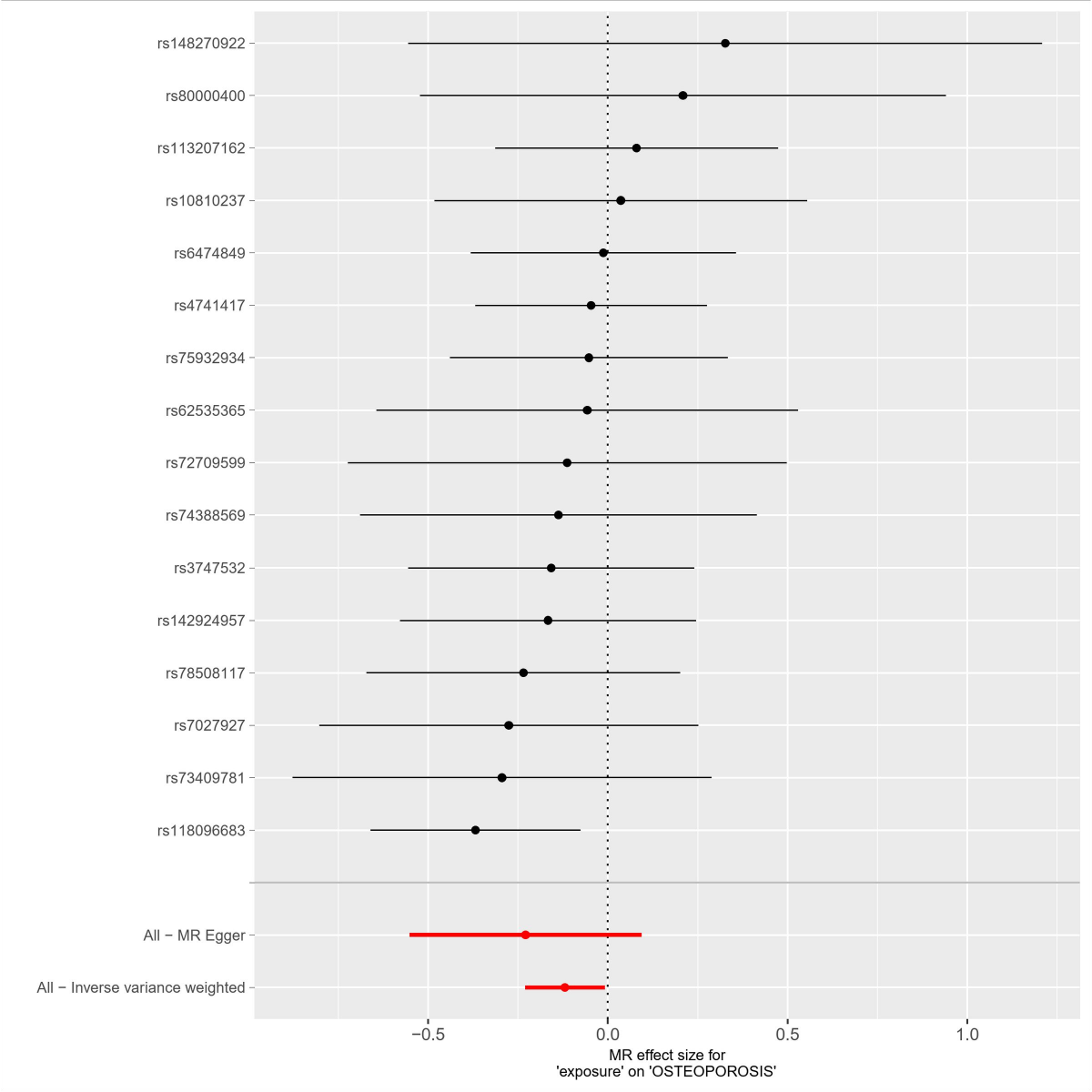

## Slide 28
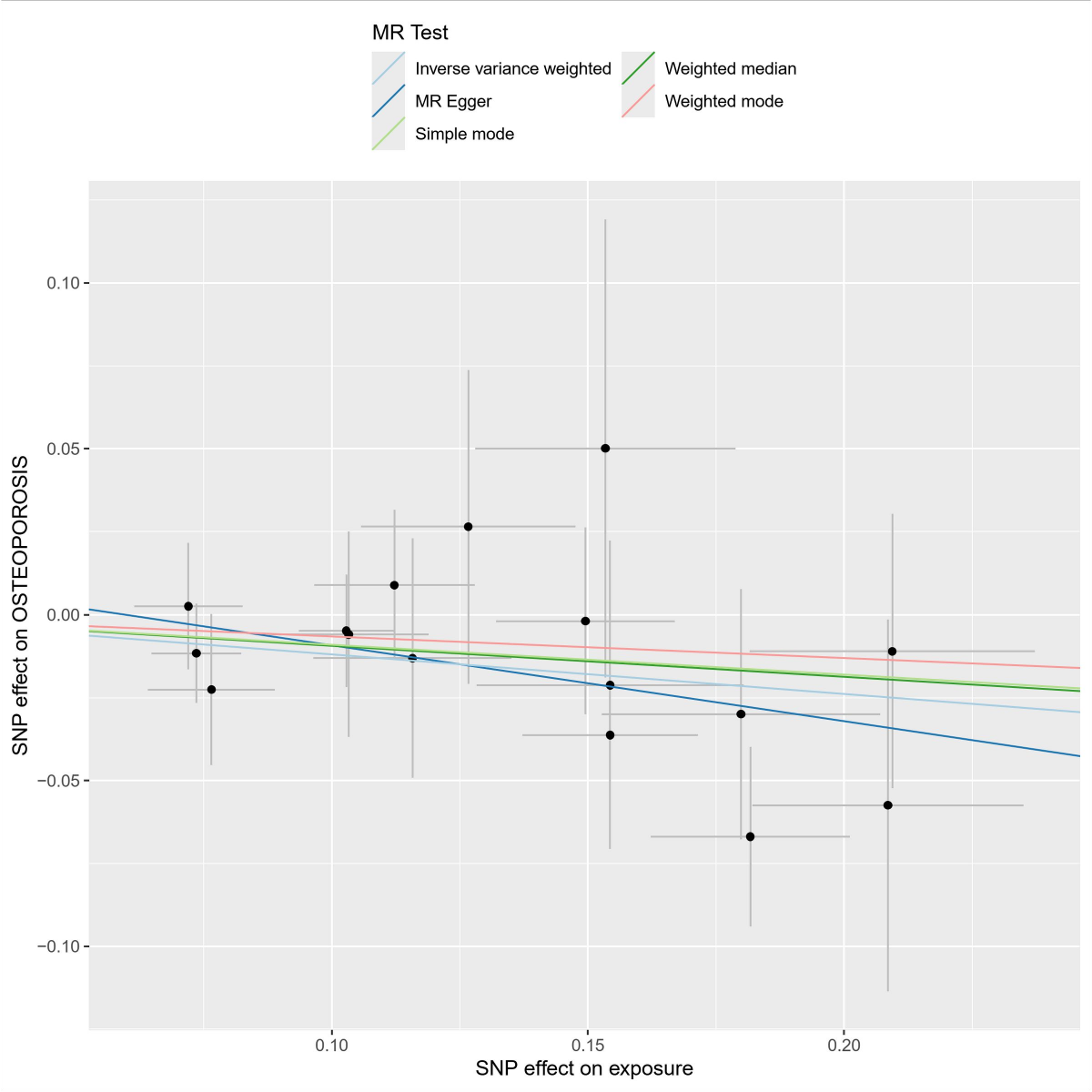

## Slide 29
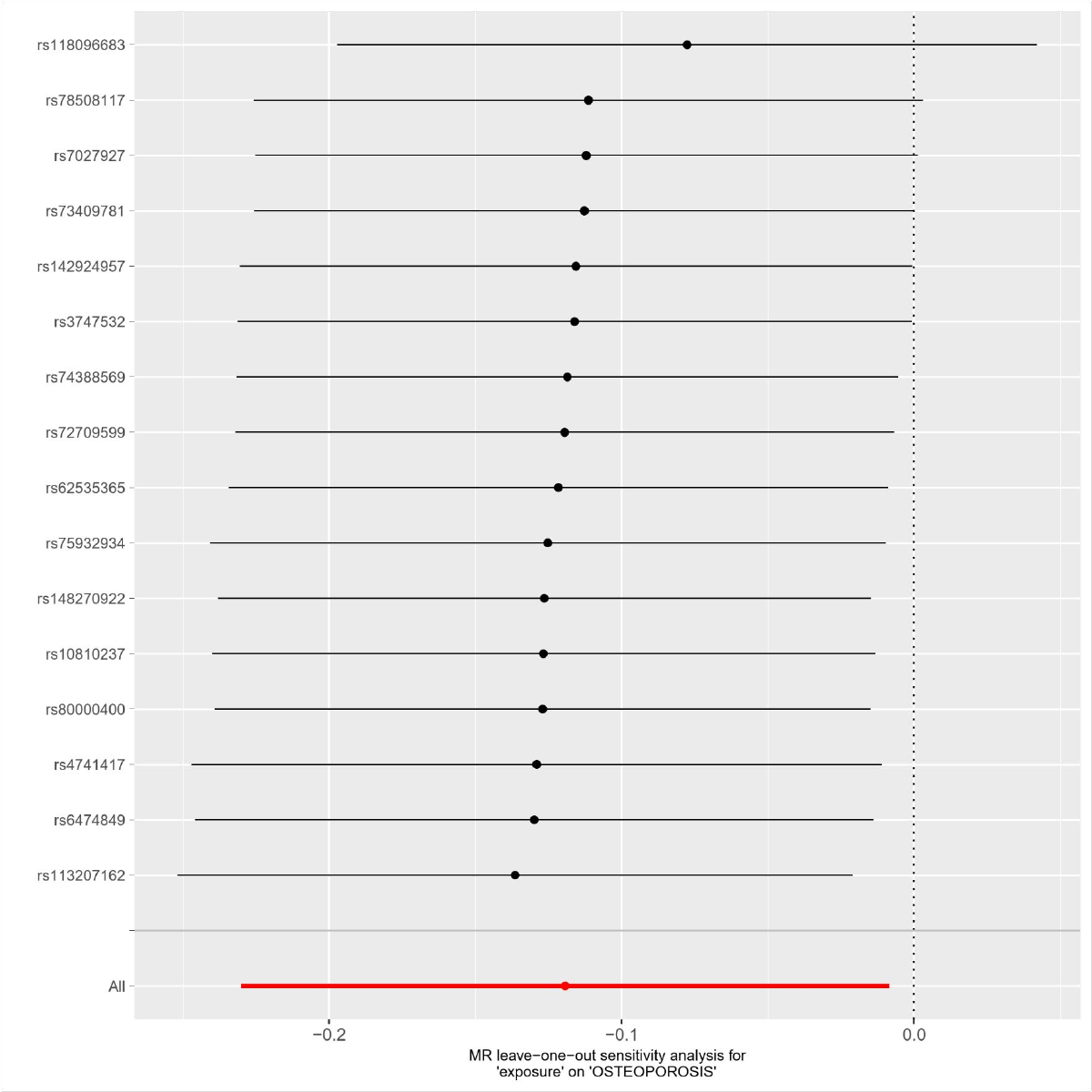

## Slide 30
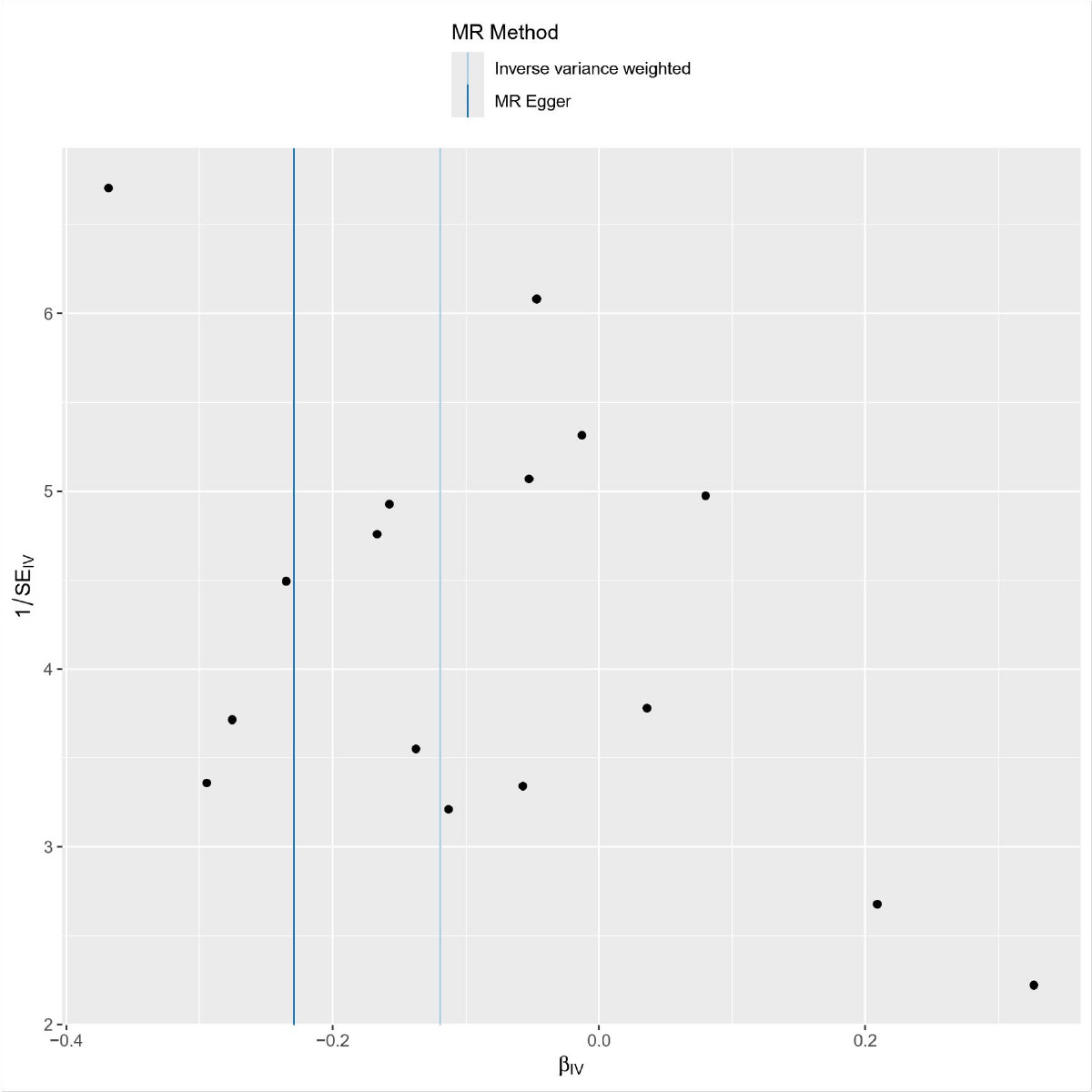

## Slide 31
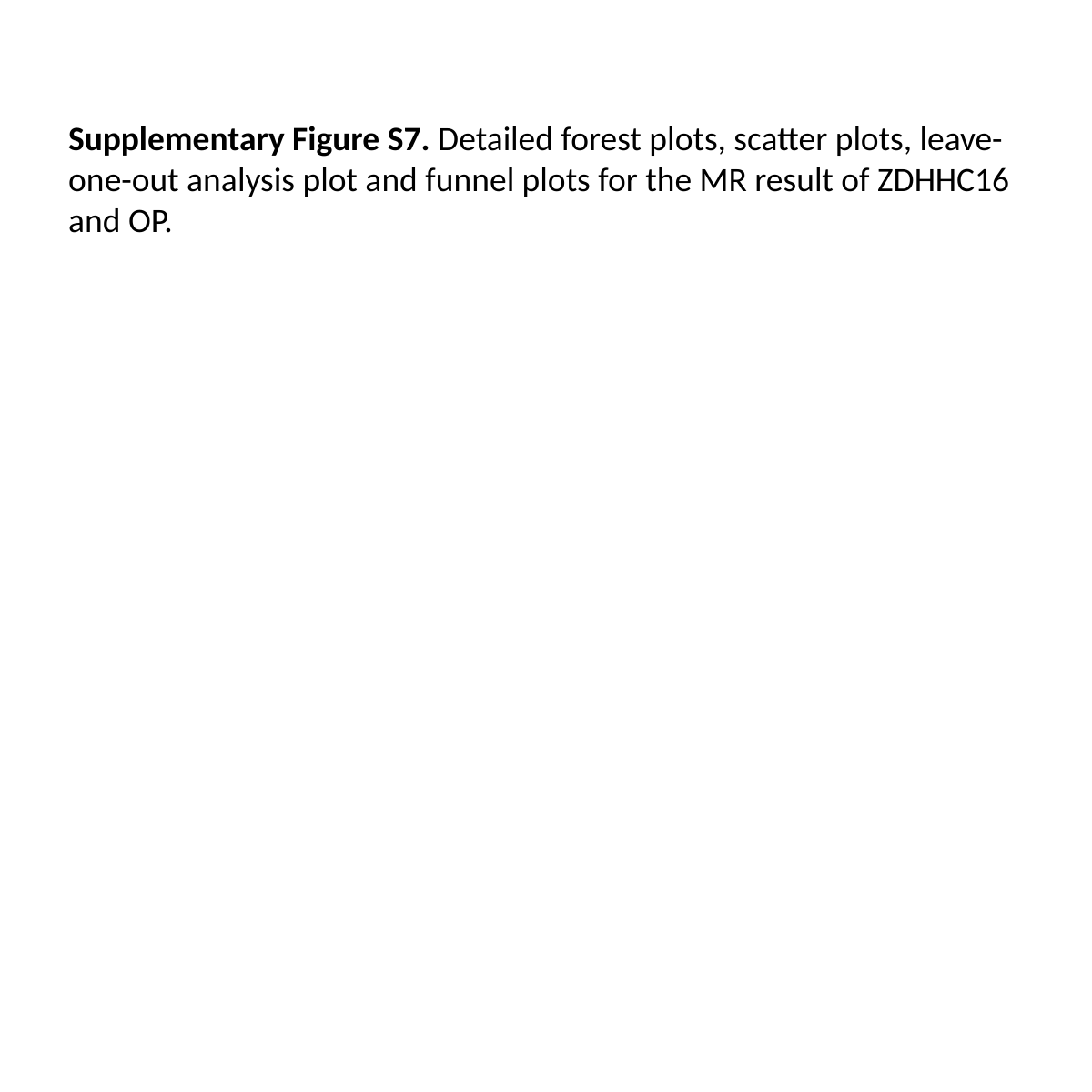

Supplementary Figure S7. Detailed forest plots, scatter plots, leave-one-out analysis plot and funnel plots for the MR result of ZDHHC16 and OP.

## Slide 32
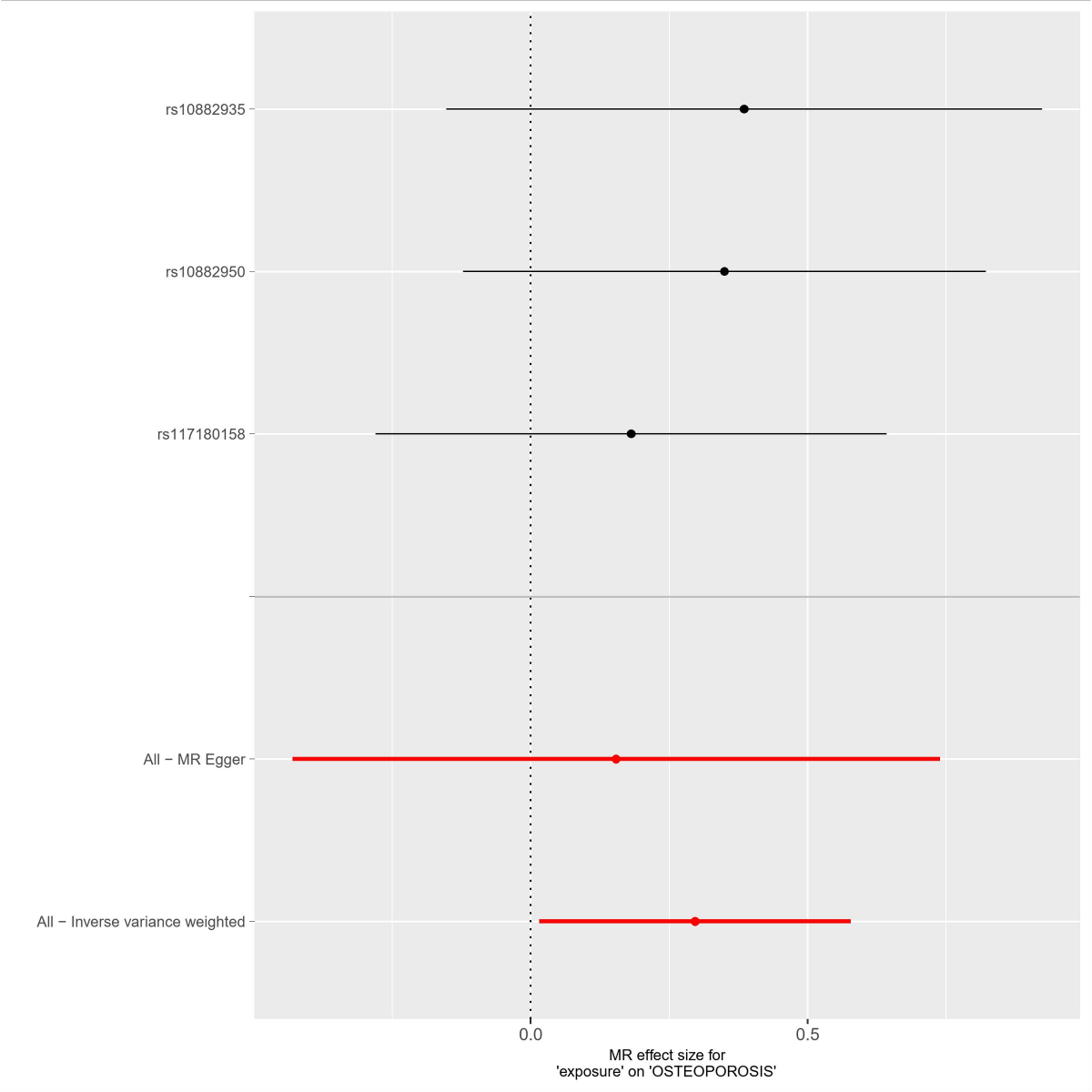

## Slide 33
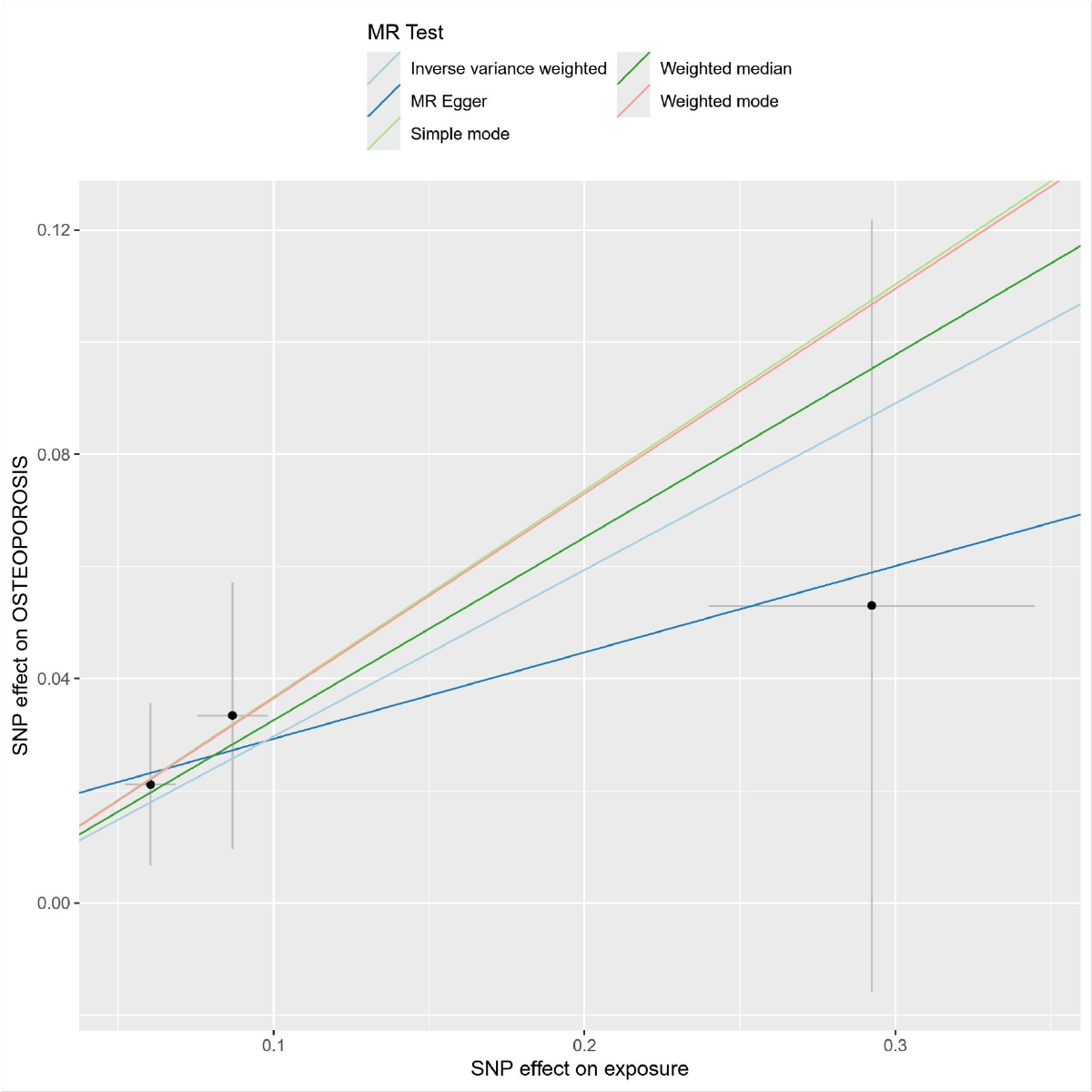

## Slide 34
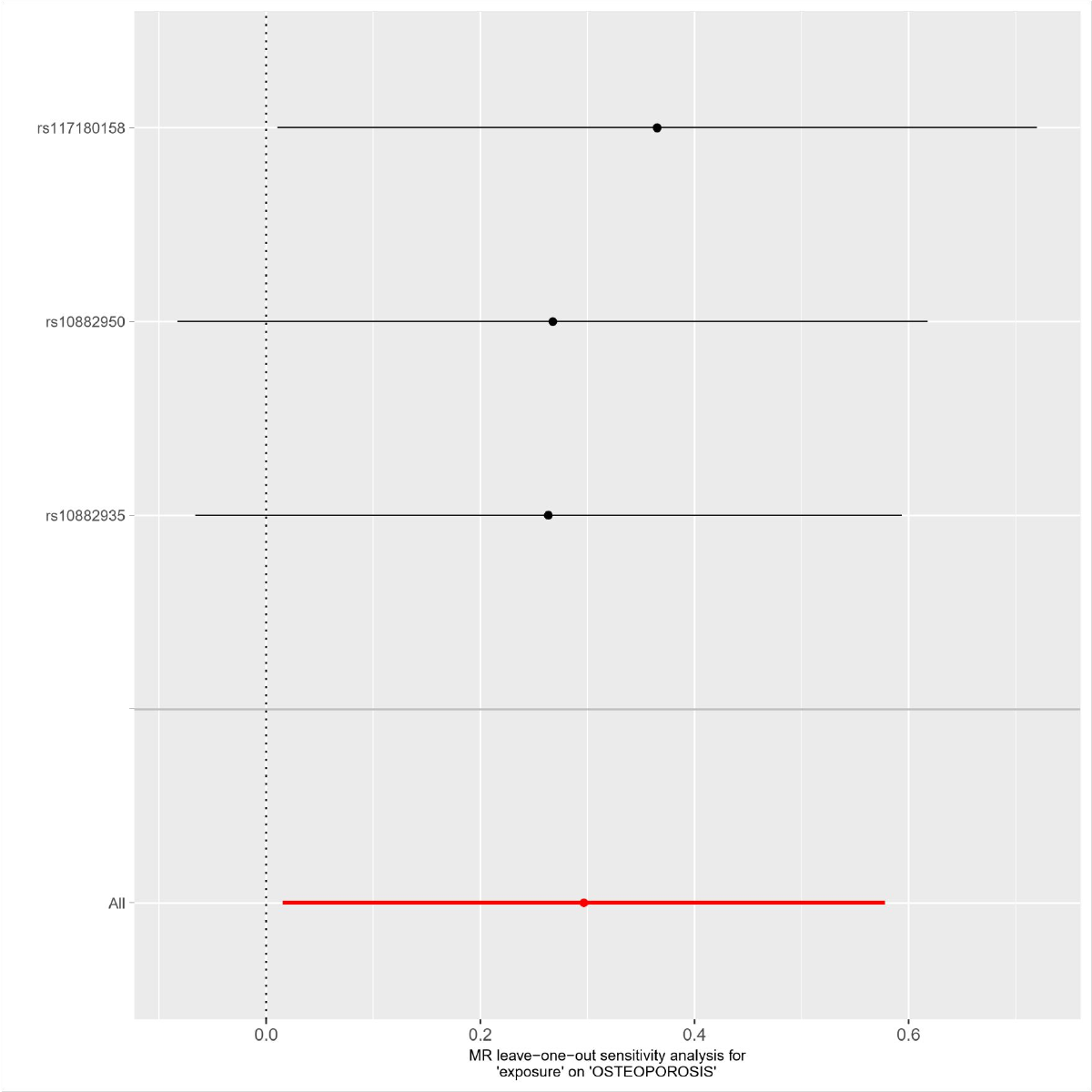

## Slide 35
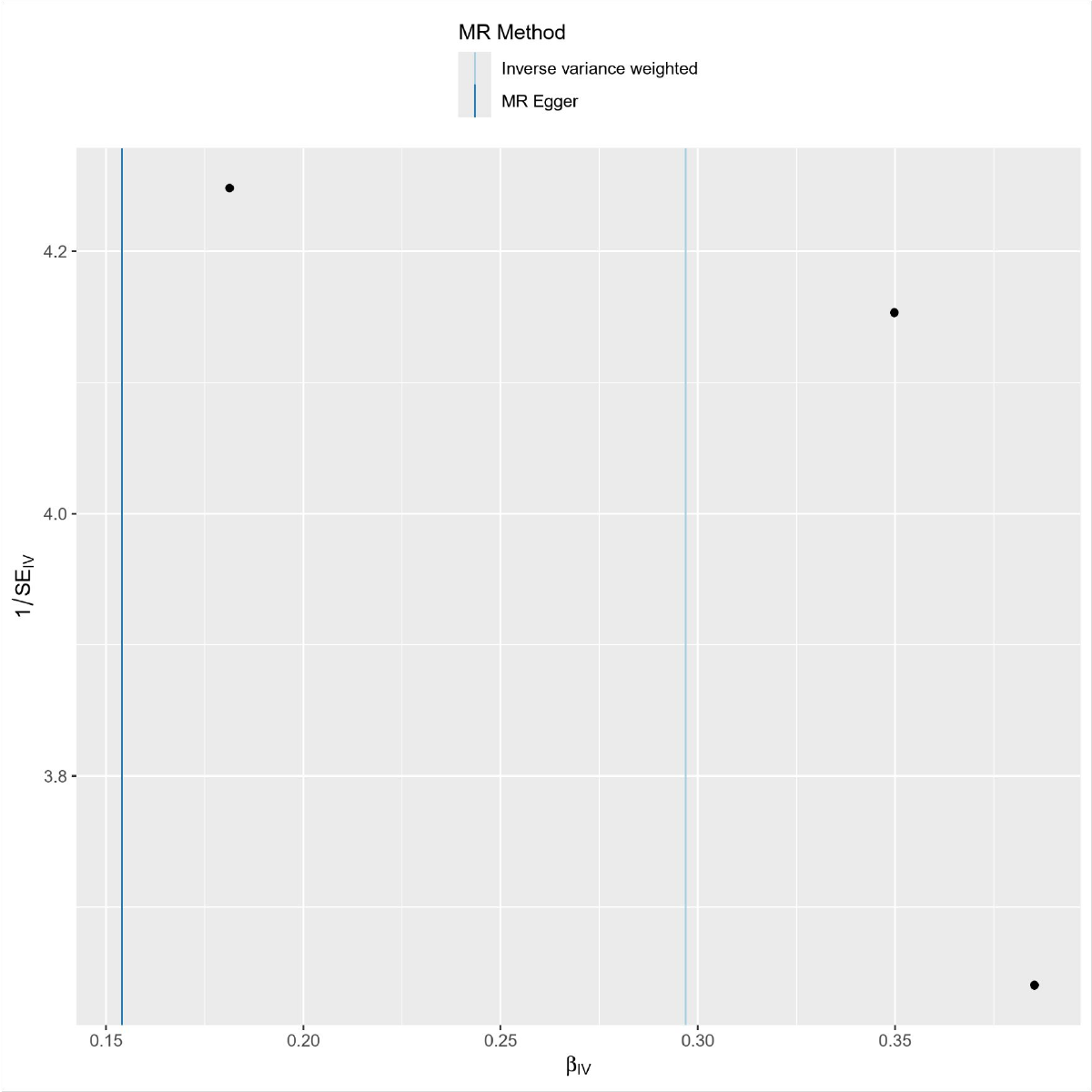

## Slide 36
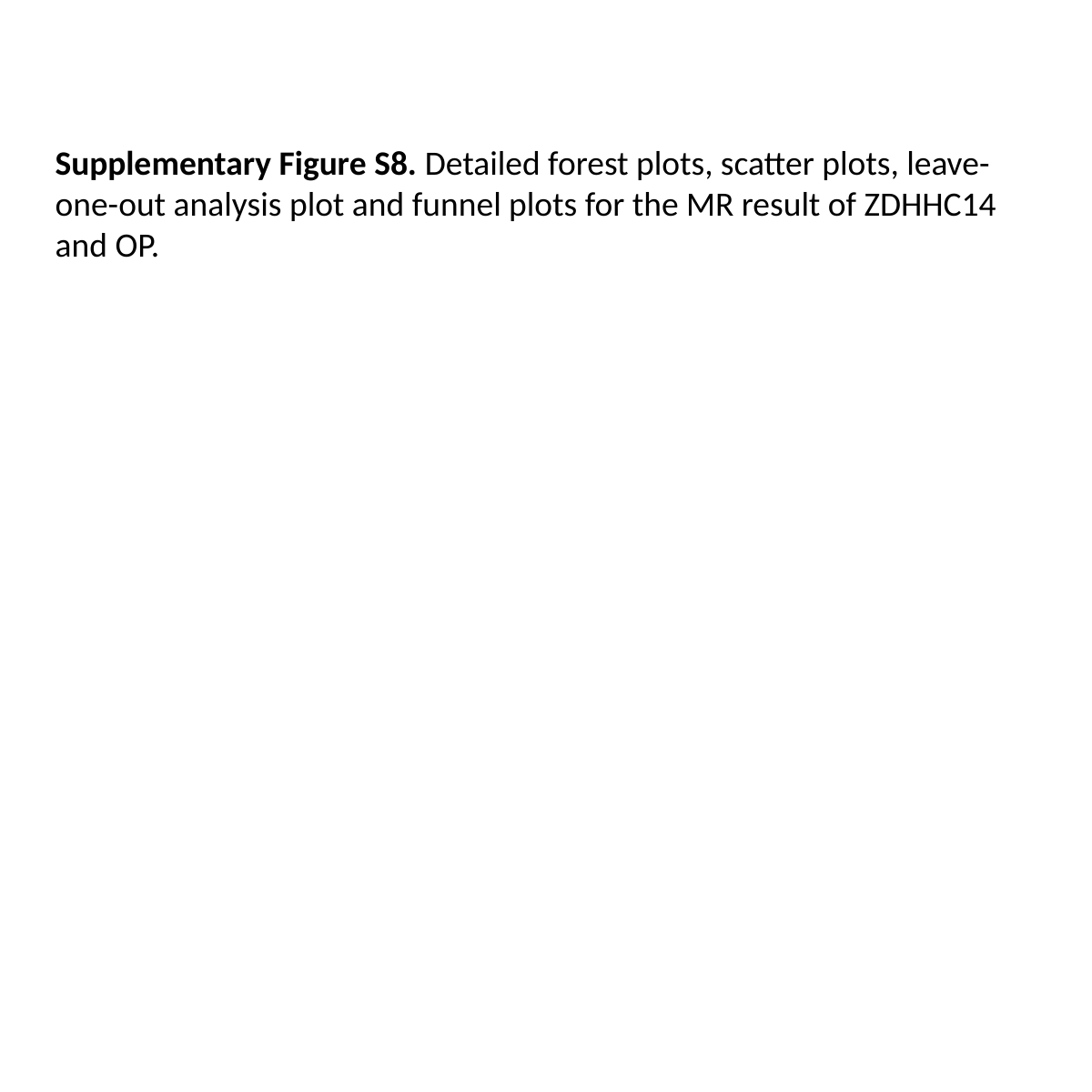

Supplementary Figure S8. Detailed forest plots, scatter plots, leave-one-out analysis plot and funnel plots for the MR result of ZDHHC14 and OP.

## Slide 37
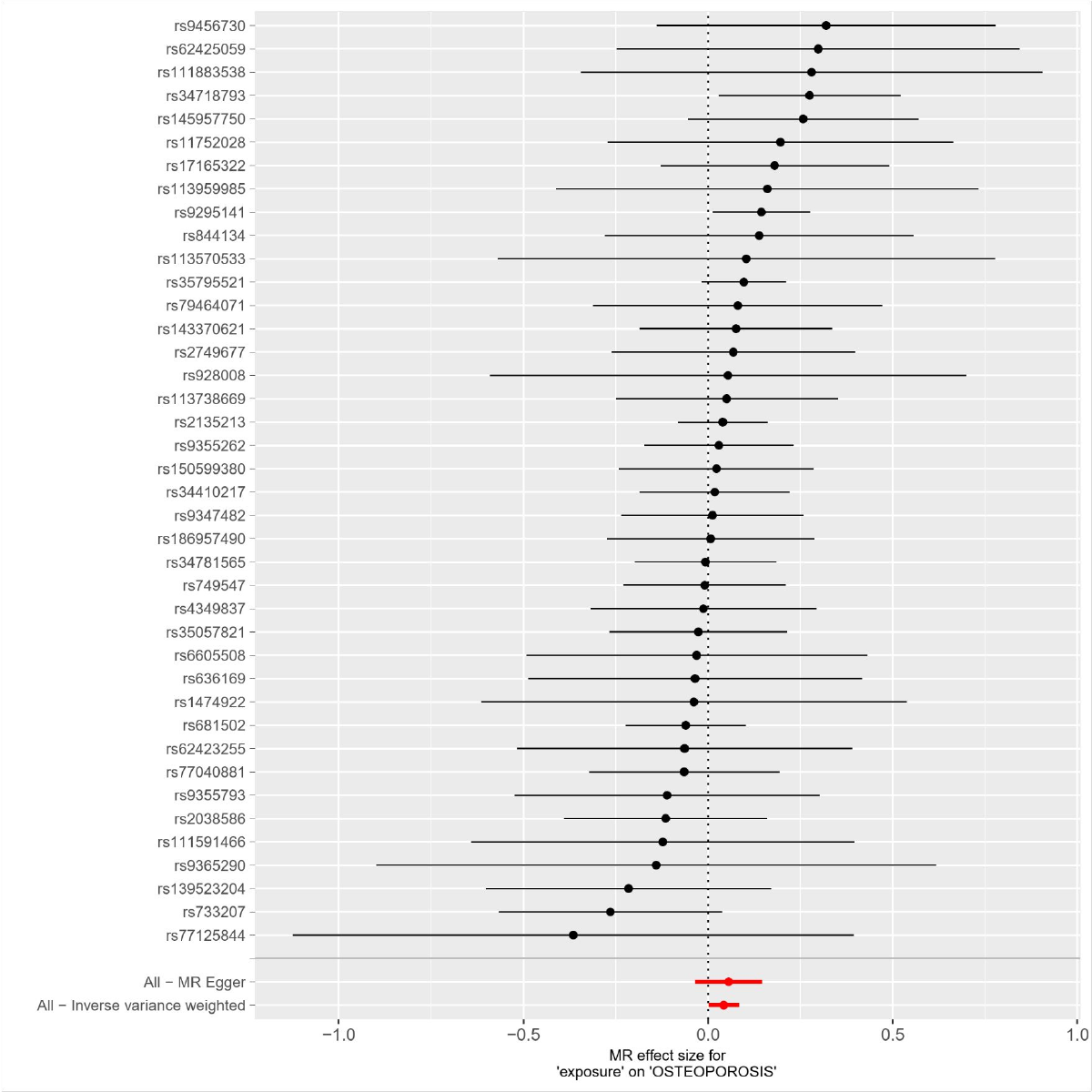

## Slide 38
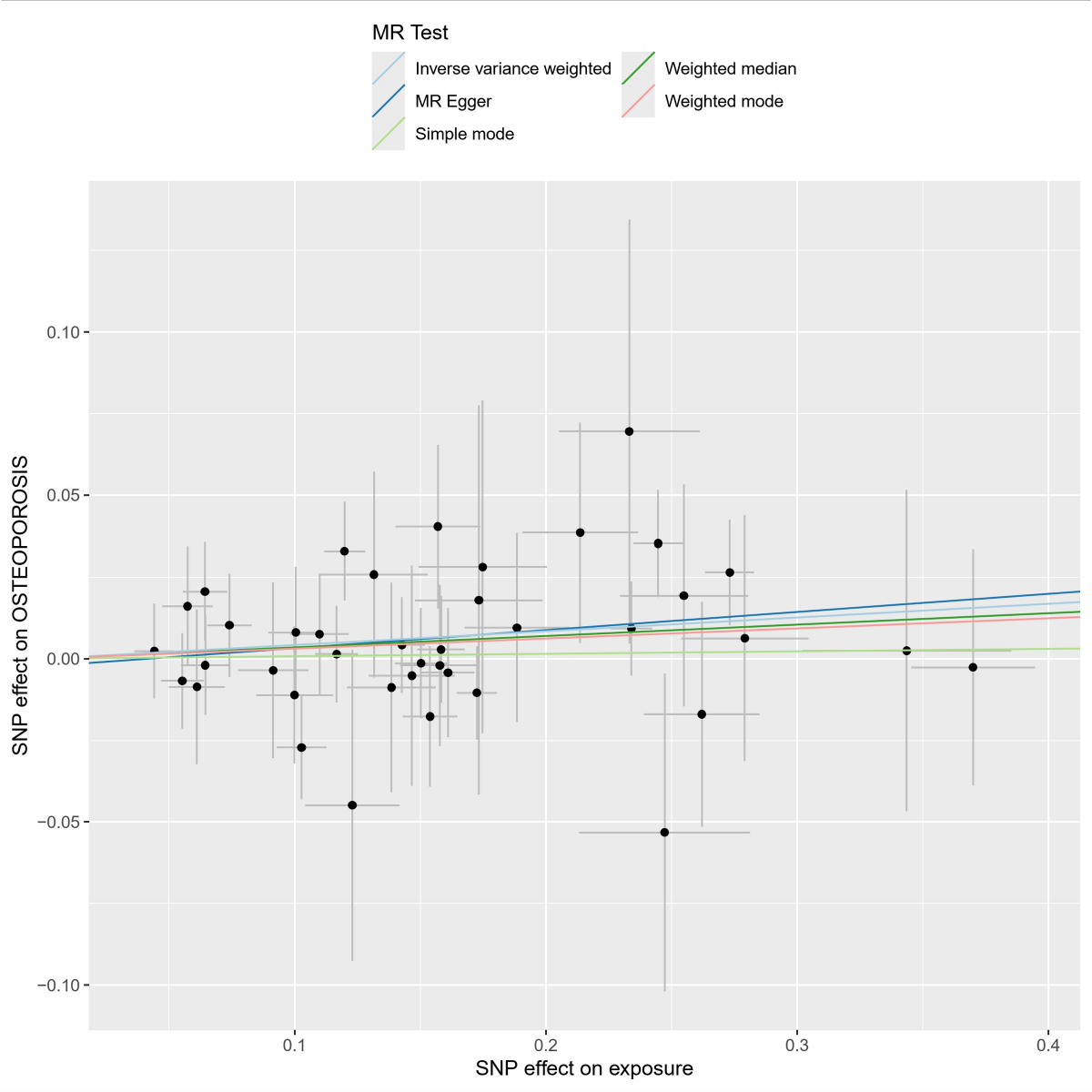

## Slide 39
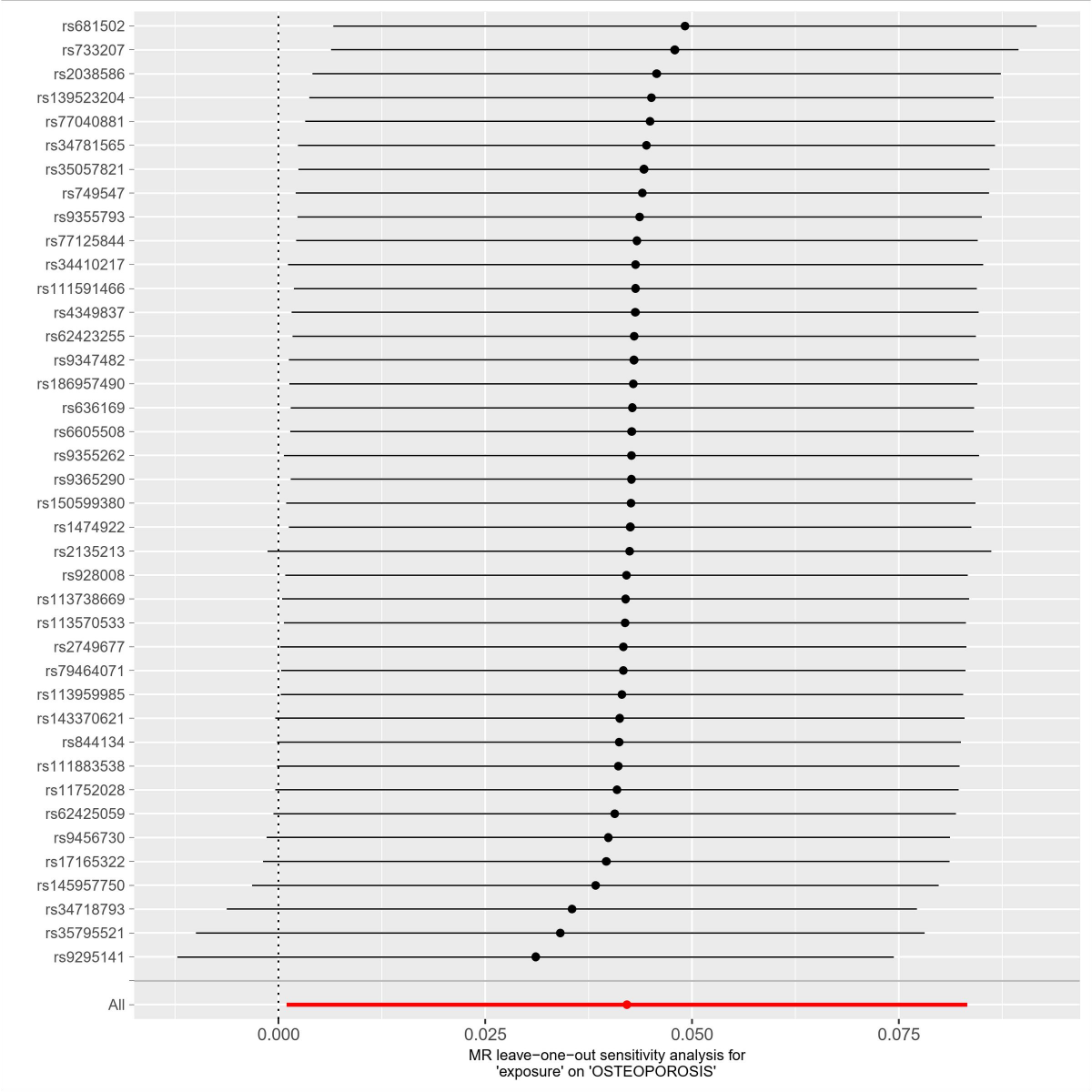

## Slide 40
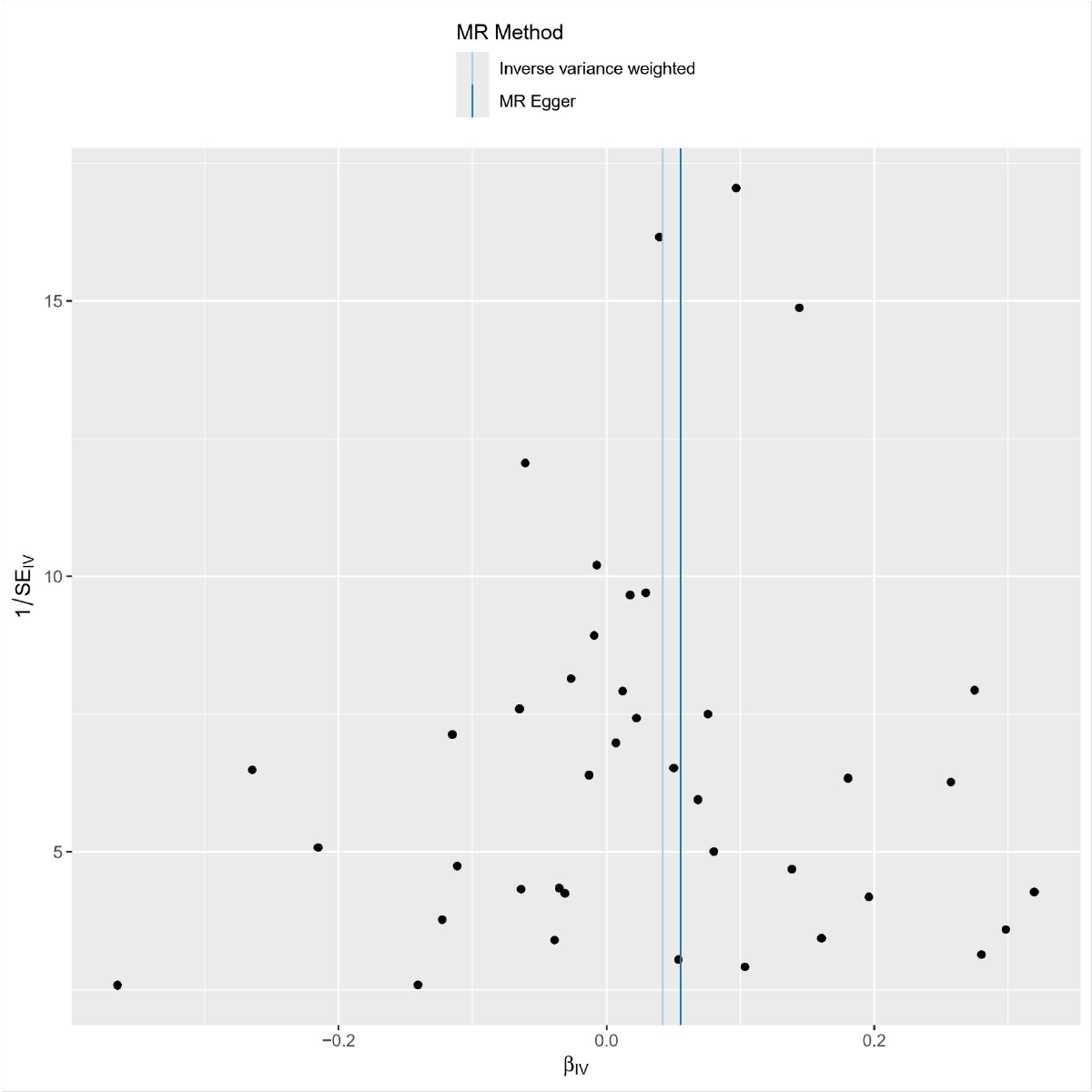

## Slide 41
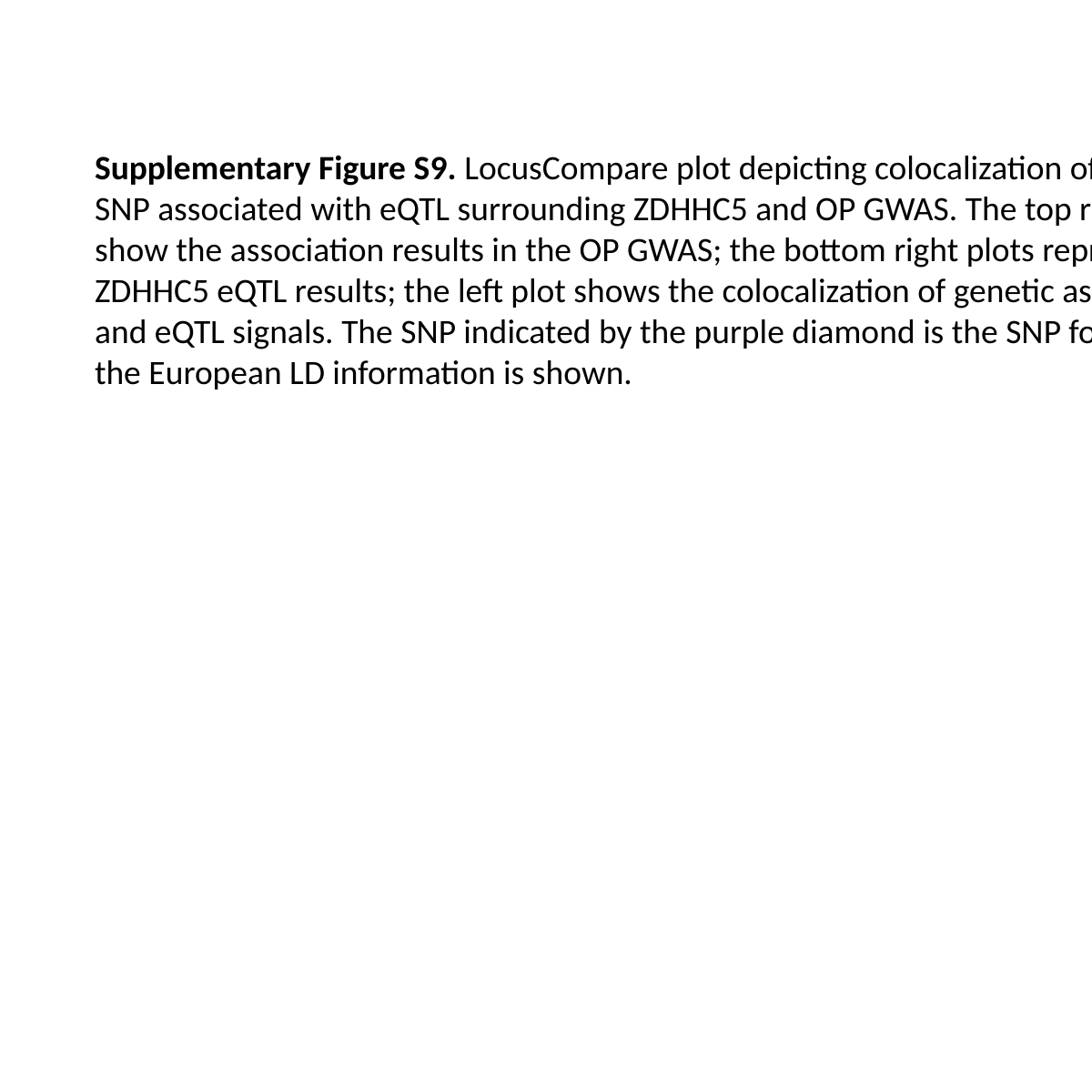

Supplementary Figure S9. LocusCompare plot depicting colocalization of the top SNP associated with eQTL surrounding ZDHHC5 and OP GWAS. The top right plots show the association results in the OP GWAS; the bottom right plots represent the ZDHHC5 eQTL results; the left plot shows the colocalization of genetic association and eQTL signals. The SNP indicated by the purple diamond is the SNP for which the European LD information is shown.

## Slide 42
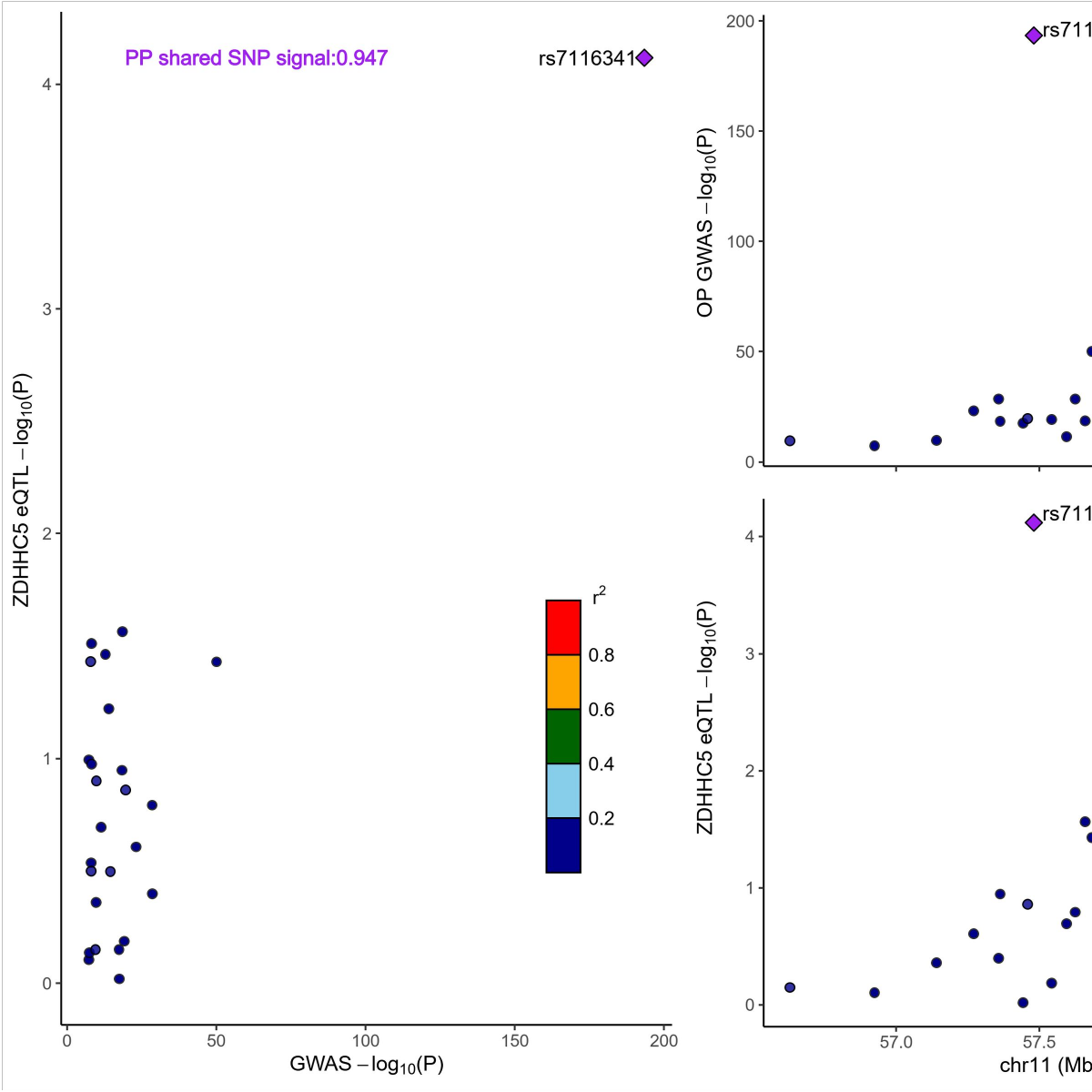

Supplement: Supplementary file 2 [file medi-105-e48429-s002.pptx]
